# What ‘Omics can tell us about antifungal adaptation

**DOI:** 10.1093/femsyr/foab070

**Published:** 2021-12-27

**Authors:** Gabriela Fior Ribeiro, Eszter Denes, Helen Heaney, Delma S Childers

**Affiliations:** University of Aberdeen, Institute of Medical Sciences, Aberdeen Fungal Group, Aberdeen, UK, AB25 2ZD; University of Aberdeen, Institute of Medical Sciences, Aberdeen Fungal Group, Aberdeen, UK, AB25 2ZD; University of Aberdeen, Institute of Medical Sciences, Aberdeen Fungal Group, Aberdeen, UK, AB25 2ZD; University of Aberdeen, Institute of Medical Sciences, Aberdeen Fungal Group, Aberdeen, UK, AB25 2ZD

**Keywords:** 'Omics, *Candida albicans*, *Candida glabrata*, *Saccharomyces cerevisiae*, antifungals, resistance

## Abstract

Invasive candidiasis, the most frequent healthcare-associated invasive fungal infection, is commonly caused by *Candida albicans*. However, in recent years other antifungal-resistant *Candida* species—namely *Candida glabrata* and *Candidaauris*—have emerged as a serious matter of concern. Much of our understanding of the mechanisms regulating antifungal resistance and tolerance relies on studies utilizing *C. albicans, C*. *glabrata*and the model yeast *Saccharomyces cerevisiae*. ‘Omics studies have been used to describe alterations in metabolic, genomic and transcriptomic expression profiles upon antifungal treatment of fungal cells. The physiological changes identified by these approaches could significantly affect fungal fitness in the host and survival during antifungal challenge, as well as provide further understanding of clinical resistance. Thus, this review aims to comparatively address ‘omics data for *C. albicans, C. glabrata andS. cerevisiae* published from 2000 to 2021 to identify what these technologies can tell us regarding cellular responses to antifungal therapy. We will also highlight possible effects on pathogen survival and identify future avenues for antifungal research.

## Teaser

‘Omics technologies provide vital insights into the physiological adaptations that occur in response to antifungals, but key gaps remain in understanding antifungal adaptation in model yeast and pathogenic fungi.

## INTRODUCTION

Invasive candidiasis is a life-threatening fungal disease that is most often caused by *Candida albicans*. The incidence of invasive candidiasis is estimated at ∼5100 individuals per year in the UK and ∼25 000 patients per year in the USA, and in particular affects patients on intensive chemotherapy, immunosuppressive drugs or long-term hospital stays (Bongomin *et al*. 2017; Pegorie, Denning and Welfare 2017; Tsay *et al*. 2020). However, recent increases in the clinical incidence of other non-*albicans* species have been observed, particularly for antifungal-resistant species (Guinea 2014; Lamoth *et al*. 2018; Ricotta *et al*. 2020). This increasing frequency of infections by drug and multi-drug resistant fungal pathogens presents a complex modern clinical challenge that requires urgent attention.

*Candida glabrata* and *Candida auris* are two important species of concern that can cause drug-resistant candidiasis. *Candida glabrata* is the second leading cause of invasive candidiasis in several geographical regions, including North America and Europe (Bongomin *et al*. 2017; Ricotta *et al*. 2020). *Candida glabrata* rapidly acquires resistance to azoles and can develop resistance to a second drug class, the echinocandins (Healey and Perlin 2018). *Candida auris* is an emerging fungal pathogen that is classified as a serious global health threat by the Centers for Disease Control due to its alarming rates of multi-drug resistance, with some isolates resistant to all three major antifungal drug classes (CDC 2021). Since its first description in 2009 (Satoh *et al*. 2009), *C. auris* has caused multiple hospital outbreaks prompting calls for improved diagnostics and renewed efforts for antifungal development. However, we currently do not know how antifungal resistance mechanisms exhibited by these species affect transmission, commensalism and other aspects of host–pathogen interactions.

Drug susceptibility can be quantified *in vitro* by minimum inhibitory concentration (MIC) testing. Antifungal resistance is usually defined as the acquisition of genetic mutations within a population that confers the ability to grow at high MICs. Antifungal tolerance permits growth in the zone of inhibition, but is distinct from resistance. Tolerant sub-populations can proliferate slowly at high azole MICs and survive lethal echinocandin challenge without acquiring adaptive genetic mutations or altering the MIC of the population (Robbins, Caplan and Cowen 2017; Healey and Perlin 2018; Rosenberg *et al*. 2018). However, little is known about the mechanisms that drive tolerant adaptation versus antifungal resistance.

**Figure 1.**
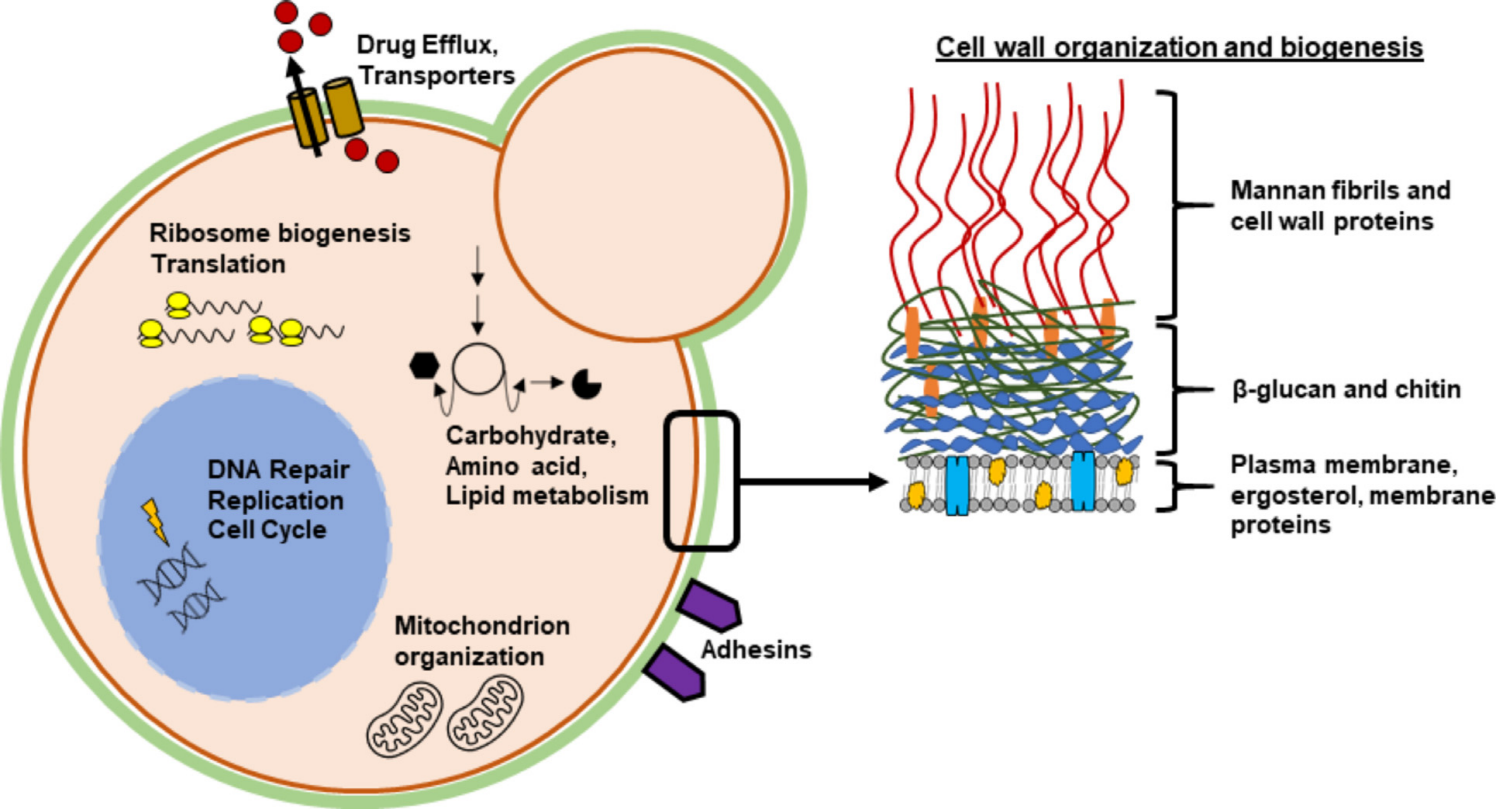
Common biological processes affected by antifungal treatment. This yeast cell diagram highlights GO Biological Processes that were frequently differentially regulated in antifungal ‘omics studies.

**Figure 2.**
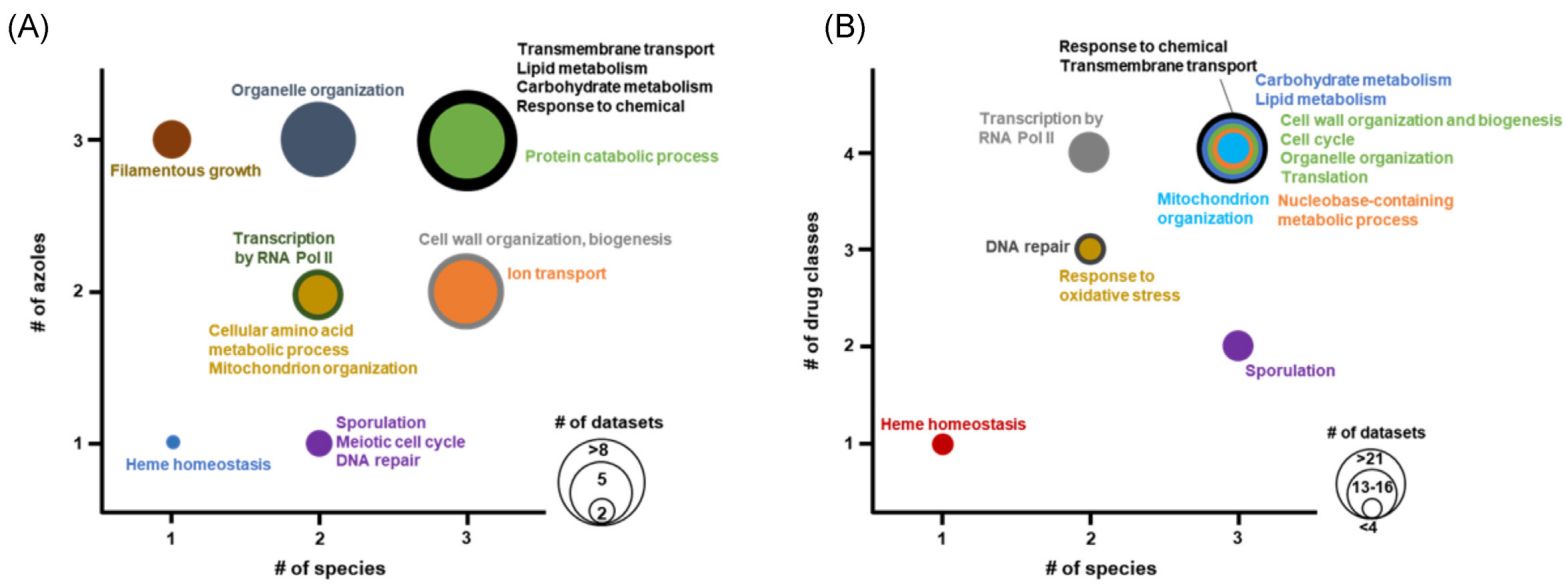
Bubble chart for the top recurrent GO Biological Process terms across antifungal ‘omics datasets. Publicly available datasets from the articles listed in supplemental data were analyzed for GO Slim Biological Processes on SGD and CGD. Bubble size scales with the number of datasets where the GO Biological Process was differentially expressed in antifungal treated versus control cells. **(A)** Differentially regulated GO Biological Processes for *S. cerevisiae*, *C. glabrata* and *C. albicans* transcriptomic and proteomic datasets featuring fluconazole, miconazole or ketoconazole treated cells. **(B)** A subset of the top differentially regulated GO Biological Processes for *S. cerevisiae*, *C. glabrata* and *C. albicans* transcriptomic, and proteomic datasets for all four drug classes (i.e. 5-flucytosine, amphotericin B, caspofungin and azole drugs). Note: panel B bubble scale is determined by the number of datasets in multiples of 4 (i.e. 1–4, 5–8, 9–12, 13–16, 17–20 and 21–24 datasets).

Much of our current understanding of the molecular mechanisms underlying antifungal resistance or tolerance in *Candida* spp. relies on work undertaken in *C. albicans, C. glabrata* and the model yeast, *Saccharomyces cerevisiae*. Interestingly, many antifungal resistance mechanisms are conserved between these organisms, and these will be discussed further in this review. In addition to these canonical antifungal resistance mechanisms, ‘omics studies have highlighted massive changes in metabolic flux and gene and protein expression profiles when cells are stressed by antifungals (Figs 1 and 2). These alterations in physiological processes are poorly understood, but could ultimately affect host fitness and survival during antifungal challenge and provide greater insight into clinical resistance. Therefore, this review will address what ‘omics data from *S. cerevisiae, C. albicans* and *C. glabrata* can tell us about cellular responses to antifungal therapy and highlight how these responses may affect pathogen survival during commensalism or infection.

## OVERVIEW OF MOLECULAR ANTIFUNGAL RESISTANCE MECHANISMS

Clinical resistance is defined as infection persistence due to a failure to inactivate or kill fungal pathogens despite appropriate treatment (Kanafani and Perfect 2008). Clinical resistance is not always correlated with *in vitro* resistance, which is often measured as the MIC of a given drug. There is speculation that this lack in correlation is due to, perhaps in part, the multivariate nature of clinical resistance, which relies on the interaction between the pathogen, the host and the pharmacokinetics of the chosen drug.

The antifungal drugs currently used in the clinic for invasive disease can be divided into four classes based on their mechanism of action: azoles, echinocandins , polyenes and flucytosine (5-flucytosine, 5-fluorocytosine, or 5-FC). Major mechanisms for resistance to these drugs are listed in Table 1 and briefly summarized below (and addressed in more detail in these reviews; Sanglard, Coste and Ferrari (2009), Cowen *et al*. (2014) and Bhattacharya, Sae-Tia and Fries (2020)). Treatment options for fungal infections remain limited largely because many compounds, although effective, are extremely toxic to mammalian cells due to similarities between host and fungal cell biology. These therapeutic limitations underscore the risks of emerging antifungal resistance and the need for continued development of new antifungals.

**Table 1.** Key mechanisms and genes involved in antifungal resistance.

	*S. cerevisiae*	*C. glabrata*	*C. albicans*
**Azoles**			
Ergosterol biosynthesis upregulation via	√	√	√
• *ERG3/6* loss of function mutation	*ERG3/6/11*	*ERG11*	*ERG3/11*
• *ERG11* or *UPC2* gain-of-function mutations or overexpression	*UPC2*	*UPC2A*	*UPC2*
Increased efflux pump activity via	√	√	√
• *PDR1, MRR1* or *TAC1* gain-of-function mutations	*PDR1*	*PDR1*	*TAC1*and*MRR1*
• *CDR1/2* overexpression	*PDR5*	*CDR1/2*	*CDR1/2*
Increased ABC transporter activity via	√	√	√
• *STB5* loss of function mutation		*STB5*	*STB5*
• *SNQ2*, *PDH1* or *YOR1* overexpression	*SNQ2*	*SNQ2*and*PDH1*,	*YOR1*
		*YOR1*	
Increased drug:H + antiporter activity via	√	√	√
*AQR1, FLR1*, *QDR2, TPO1_1* or *MDR1* overexpression	*AQR1*and*FLR1*	*QDR2*and*TPO1*	*MDR1*
**Echinocandins**			
Modification of the glucan synthase enzyme or its activity	√		
• *FKS1* and *FKS2* mutations	*FKS1/2*	*FKS1/2*	*FKS1/2*
• *SBE2* overexpression	*SBE2*		
Long chain base accumulation (CRIS-MIS), *SUR2* and *FEN1* loss of function	√	√	√
Disturbance in programmed cell death, *AIF1* loss of function mutation	√	√	√
Increased chitin levels (*CHS* mutations)	√	√	√
**Polyenes**			
Depleted ergosterol from the cell membrane, *ERG1/2/3/5/6/11* loss of function	√	√	√
	*ERG3/6*	*ERG1/2/6/11*	*ERG3/5*
Reduced ribosome synthesis, *TORC1* loss of function	√	√	√
Increased reactive oxygen species	√	√	√
Detoxification, *SOD3*, *RAS1/2, TOR1* or *BSC2* mutations	*RAS1/2, TOR1*and*BSC2*		*SOD3*
**5-FC**			
Decreased enzyme activity preventing the conversion chain of 5-FC into fungistatic	*FUR1*and*FCY1/2*	*FUR1*and*FCY1/2*	*FUR1*and*FCY1/2*
5-FUTP, *FUR1, FCY1/2*and*FCY21/22* loss of function mutation	*FCY21/22*		*FCY22*
Continuation of DNA synthesis despite the presence of fluorinated analogues, *CDC21* overexpression	√	√	√
Prevention of drug accumulation by hyperactive transporters or antiporters, *FLR1/2*, *CDR1* and *PDR1*	√	√	√

Several antifungal resistance mechanisms are conserved in *S. cerevisiae*, *C. glabrata* and *C. albicans*. Species-specific genes and processes are indicated where appropriate.

### Azoles

*Candida* spp. azole resistance became a clinical concern shortly after the market debut of fluconazole in the late 1980s (Smith *et al*. 1986). Azoles can be subdivided based on their chemical structures into imidazoles (e.g. ketoconazole and miconazole) and triazoles (e.g. fluconazole, itraconazole and voriconazole). Azole-resistant isolates from candidemia patients have been observed at low frequency for *C. albicans* infections (0–5%), but are frequently found in *C. glabrata* infections (11–15%; Diekema *et al*. 2012; Pfaller, Jones and Castanheira 2014; Pfaller *et al*. 2015). Azoles target and inhibit a key enzyme in the ergosterol biosynthetic pathway, lanosterol 14α-demethylase (*ERG11* in *Candida* spp.). Ergosterol is the major fungal sterol present in the plasma membrane and contributes to the permeability and fluidity of the membrane, ensures cytoskeleton organization and regulates the activity of membrane transporters (Sgherri *et al*. 2014).

Azole resistance in *S. cerevisiae*, *C. glabrata* and *C. albicans* has been linked *in vitro* to two general mechanisms—alterations in sterol metabolism or reducing intracellular drug concentrations. First, gain-of-function (GOF) mutations or alterations in the expression of genes linked to ergosterol biosynthesis, such as the azole target *ERG11* (Kontoyiannis, Sagar and Hirschi 1999; Hull *et al*. 2012a), *ERG6* (Anderson *et al*. 2003; Xu *et al*. 2007), *ERG3* (Anderson *et al*. 2003; Martel *et al*. 2010), or the sterol metabolism transcriptional regulator *UPC2* (Dunkel *et al*. 2008a; Whaley *et al*. 2014), can render cells less sensitive to azole activity. Loss of function mutations in *ERG3* initiate a metabolic bypass that prevents the accumulation of toxic sterol intermediates, which renders cells less susceptible to growth inhibition by azoles (Martel *et al*. 2010). Second, cells can limit cytoplasmic azole concentrations via upregulation of drug efflux pump expression, such as the ABC transporters *CDR1*, *CDR2* and *SNQ2*, through pump promoter mutations (Sanglard *et al*. 1995; Mahé *et al*. 1996; Torelli *et al*. 2008) or GOF mutations to the transcription factors *PDR1*, (*S. cerevisiae* and *C. glabrata*), *MRR1* (Dunkel *et al*. 2008b) or *TAC1* (*C. albicans*; Mahé *et al*. 1996; Coste *et al*. 2004; Vermitsky and Edlind 2004; Tsai *et al*. 2006). In addition to drug transporter expression, *PDR1* controls expression of *RPN4*, a transcriptional regulator of proteasomal genes that also mediates azole susceptibility in *S. cerevisiae* (Owsianik, Balzi and Ghislain 2002) and *C. glabrata* (Pais *et al*. 2020). *CgPDR1* also affects the expression of the adhesin *EPA1*, and *PDR1* GOF mutations have been associated with increased adherence to epithelial cells and enhanced virulence in mouse candidemia models (Ferrari *et al*. 2009; Vale-Silva *et al*. 2016). *Candida glabrata* clinical isolates are intrinsically less susceptible to azole drugs due to their high-level expression of drug efflux pumps (Vermitsky and Edlind 2004). Mutations in ergosterol biosynthesis genes and alterations in efflux pump expression have also been observed in drug-resistant clinical isolates (vanden Bossche *et al*. 1992; Marichal *et al*. 1999; Rogers and Barker 2003; Bennett, Izumikawa and Marr 2004; Xiang *et al*. 2013).

### Echinocandins

The echinocandins are the most recent of the four antifungal drug classes to be developed with caspofungin hitting the market in the early 2000s. Echinocandins (i.e. caspofungin, micafungin and anidulafungin) are the preferred first-line choice for treatment of invasive *Candida* infections, in part due to the increasing prevalence of azole-resistant non-*albicans Candida* species (Pappas *et al*. 2016). This drug class inhibits β-glucan synthesis leading to a loss of cell wall integrity that can be fungicidal or fungistatic. Approximately 2–3% of *C. albicans* (Castanheira *et al*. 2010) clinical isolates develop echinocandin resistance compared to 1→10% of *C. glabrata* isolates, depending on the geographical region surveyed (Perlin 2015).

Clinical and *in vitro e*chinocandin resistance in *S. cerevisiae*, *C. albicans* and *C. glabrata* is largely conferred by point mutations in the major glucan synthase enzymes, *FKS1* and *FKS2* (Douglas *et al*. 1997; Johnson, Katiyar and Edlind 2011; Pham *et al*. 2014; Suwunnakorn *et al*. 2018). These point mutations interfere with or inhibit echinocandin interactions with glucan synthase. In *C. albicans* and *S. cerevisiae*, alterations in programmed cell death due to mutations in *AIF1* also can affect echinocandin resistance (Markovich *et al*. 2004). In addition, caspofungin-treated *C. albicans* and *fks1*Δ *S. cerevisiae* strains have increased cell wall chitin content compared to untreated or wild-type cells, respectively (Markovich *et al*. 2004; Walker, Gow and Munro 2013). However, *C. glabrata* does not alter chitin content during exposure to echinocandins (Walker, Gow and Munro 2013). In *C. glabrata*, loss of *SUR2* or *FEN1* function alters echinocandin susceptibility by modulating sphingolipid interactions with Fks (Healey *et al*. 2012). A similar phenotype was described for one *C. albicans* strain out of ten tested, which suggests that this method of generating echinocandin resistance is a low-frequency event in this species (Healey *et al*. 2015).

### Polyenes

Amphotericin B (AmB) was first discovered in 1955 and put to clinical use in 1958 making it one of the oldest of the four drug classes used against invasive fungal disease. AmB binds ergosterol in the plasma membrane leading to pore formation and ultimately cell death. Clinical resistance to AmB is low for *C. albicans* and *C. glabrata* and a recent multi-site study reported no AmB resistant candidemia isolates (Toda *et al*. 2019). However, AmB also exerts cytotoxic activity against mammalian cells which can induce organ damage, especially to kidneys (Allen 2010). Mammalian toxicity can be reduced with the use of liposomal formulations (Roberts *et al*. 2015).

Like resistance to azoles, resistance to AmB has been linked to alterations in ergosterol biosynthesis. In *C. albicans*, *C. glabrata* and *S. cerevisiae*, mutations in *ERG* genes confer some protection against AmB by depleting ergosterol from the plasma membrane (Geber *et al*. 1995; Kelly *et al*. 1996; Sanglard *et al*. 2003; Vandeputte *et al*. 2008; Martel *et al*. 2010; Hull *et al*. 2012b; Kodedová and Sychrová 2015). In addition, decreased *TORC1* function confers some AmB resistance by limiting ribosome synthesis resulting in reduced cell growth rates (Bojsen *et al*. 2016). Recent work in *C. albicans* suggested that AmB induces cellular oxidative stress that plays a role in cidality (Muzafar *et al*. 2020). Thus, alterations in *SOD3* expression led to increased cell survival after drug treatment by detoxifying intracellular reactive oxygen species (ROS). In *S. cerevisiae*, AmB-resistance was linked with altered expression of *RAS1*, *RAS2* or *BSC2*, which improved ROS detoxifying activities by enhancing expression of glutathione (Bojsen *et al*. 2016; Kong *et al*. 2020). ROS detoxifying activity has not been confirmed as a major mechanism of AmB resistance in *C. glabrata*.

### Flucytosine

5-FC has been available since 1957. In fungi, 5-FC is converted by cytosine deaminase into 5-fluorouracil (5-FU), which is incorporated into RNA and other metabolites and ultimately interferes with protein translation and DNA synthesis (Polak and Scholer 1975). While initially effective, resistance to this drug is common when used alone, therefore, 5-FC is predominantly used in combinatorial treatment strategies with the above drug classes. Resistance mechanisms to 5-FC are highly conserved in *C. albicans*, *C. glabrata* and *S. cerevisiae*. All three species have demonstrated resistance with loss of function mutations to *FUR1*, *FCY1* or *FCY2*, resulting in decreased conversion of 5-FC to 5-FU (Erbs, Exinger and Jund 1997; Dodgson *et al*. 2004; Paluszynski *et al*. 2006; Edlind and Katiyar 2010). In addition, overexpression of thymidylate synthase can bypass DNA synthesis inhibition in the presence of drug (Vandeputte *et al*. 2011). Mutations in *FCY21/22*, the purine–cytosine permease, in *S. cerevisiae* or *C. albicans* inhibits uptake of 5-FC into the cell (Hope *et al*. 2004; Paluszynski *et al*. 2006). Finally, expression of drug efflux pumps and antiporters in *C. glabrata*, including increased expression of *FLR1*, *CDR1* and the transcription factor *PDR1*, confers some resistance to 5-FC (Steier *et al*. 2013; Pais *et al*. 2016a).

### Other mechanisms involved in drug resistance

Invasive pathogens have a variety of physiological responses that allow them to adapt to otherwise toxic conditions and thus exhibit mechanisms of resistance to antifungals. First, the formation of biofilms—an association of cells enveloped by extracellular matrix (ECM) which provides protection against the external environment (Uppuluri *et al*. 2011; Ramage *et al*. 2012)—reduces antifungal diffusion to fungal cells. Consequently, the MIC values required to inactivate biofilm cells were higher when compared to non-biofilm forming isolates or planktonic-grown cells (Chandra *et al*. 2001; Mukherjee *et al*. 2003). In 2001, Chandra *et al*. demonstrated that as biofilms matured the MIC concentrations for distinct antifungal classes also gradually increased for *C. albicans*, with MICs for fluconazole and voriconazole increasing by as much as 6-fold when comparing 72 h biofilms with the initial 2 h time point. Besides an intricate ECM–cell interaction, biofilms express higher levels of efflux pumps and exhibit altered metabolic states, which further contributes to reduced drug susceptibility (Chandra *et al*. 2001; Mukherjee *et al*. 2003; Ramage *et al*. 2012). Comparatively, for *S. cerevisiae*, Bojsen, Regenberg and Folkesson (2014) observed that the response of developing biofilms to antifungals was similar to the response of exponentially growing cells. This similarity was also observed between mature biofilm and non-growing planktonic yeast cells (Bojsen, Regenberg and Folkesson 2014). These results suggest that the effects of antifungals were independent of biofilm or planktonic growth in *S. cerevisiae*. Further, Bojsen *et al*. (2016) suggested that *C. glabrata* mature biofilm drug susceptibility was similar to *S. cerevisiae*, however a gradual assessment of the response of biofilm or planktonic yeast cells to antifungals was not performed for *C. glabrata*.

In addition, Hsp90 is a molecular chaperone that plays an integral role in echinocandin resistance *in vitro* via its regulatory role in the cell wall integrity pathway. Hsp90 modulates the stability of key members of the Protein Kinase C (PKC) pathway (Leach *et al*. 2012). In response to cell wall damage, the PKC pathway triggers the phosphorylation of Slt2 (whose respective yeast homolog is Mkc1 in *C. albicans*) which initiates the Mitogen Activated Protein (MAP) kinase signaling cascade to activate downstream targets (Leach *et al*. 2012). These downstream targets include cell wall-associated genes such as chitin biosynthesis enzymes, whose role in increasing cell wall chitin content correlates with improved fungal survival in response to echinocandin treatment (Reinoso-Martin *et al*. 2003; Cota *et al*. 2008; Walker, Gow and Munro 2013). In *S. cerevisiae*, deletion of *SLT2*, *BCK1*, *PKC1* or *FKS1* results in caspofungin hypersensitivity (Reinoso-Martin *et al*. 2003). Functional genomic screening of two *C. albicans* mutant libraries (covering approximately 45% of the genome) indicated that three of the nine genes identified as being involved in modulating echinocandin resistance and tolerance are components of the PKC cell wall integrity cascade *(PKC1, SWI4*and *MKC1*; Caplan *et al*. 2018). Upon further testing of the Pkc1-MAPK pathway, Caplan *et al*. (2018) observed that Hsp90 is necessary for maintaining the stability of *C. albicans* Pkc1 and Bck1, thus allowing for the development of Hsp90-regulated echinocandin resistance as a possible mechanism to compensate for the altered expression of *FKS1*. This Hsp90-dependent echinocandin resistance, mediated by calcineurin, has also been observed in *C. glabrata* clinical isolates (Singh-Babak *et al*. 2012). More specifically, in *C. glabrata* caspofungin-induced *FKS2* is dependent on calcineurin and Hsp90, and this mechanism can be pharmacologically inhibited to limit basal tolerance and confer echinocandin susceptibility in clinical isolates. Hsp90 is also important in stabilizing calcineurin in *S. cerevisiae* and enables calcineurin-dependent responses to drug-induced cellular stresses; however, Hsp90 does not appear to modulate echinocandin susceptibility in this yeast (Singh *et al*. 2009; Singh-Babak *et al*. 2012).

*Candida albicans* resistance to the polyene AmB has been linked to Hsp90. However, Vincent *et al*. (2013) observed that AmB-resistant strains were hypersensitive to Hsp90 inhibitors due to high levels of Hsp90 function in cells even in the absence of AmB. This finding has been speculated to be the result of significant costs to fungal pathogenicity in AmB resistant strains, which includes hypersensitivity to host immune defenses and inability to invade host tissue (Vincent *et al*. 2013). Therefore, the virulence costs because of reduced susceptibility to AmB seems to lead to an evolutionary impasse, making it unfavorable for fungal cells to present AmB resistance in the clinic. The relevance of AmB resistance and the involvement of Hsp90/calcineurin in this process for *C. glabrata* and *S. cerevisiae* requires further study.

Finally, mitochondrial alterations and activation of stress pathways are also mechanisms utilized by pathogenic fungi to acquire resistance against antifungal agents. For example, loss of mitochondrial function, such as in petite mutants, in *C. glabrata* leads to increased fluconazole resistance (Sanglard, Ischer and Bille 2001). Petite mutants have elevated expression of drug efflux pumps, such as *PDR5* and *CDR1* (Brun *et al*. 2004; Demuyser *et al*. 2017). Overexpression of *MGE1*, a yeast chaperone involved in the mitochondrial protein import system, also suppresses fluconazole susceptibility in *S. cerevisiae* and *Candida* species (Demuyser *et al*. 2017).

## GENOMIC ALTERATIONS INVOLVED IN ANTIFUNGAL RESISTANCE

Besides alterations in cell structure, metabolism and membrane homeostasis, antifungals can induce significant genomic changes in fungal cells. This section will explore what we know about antifungal-induced genomic plasticity in *C. albicans, C. glabrata* and *S. cerevisiae*.

### The role of mating, aneuploidy and isochromosomes in antifungal adaptation

Mating is a mechanism for generating genetic diversity and can be induced by antifungal stress in *C. albicans* (Rustad *et al*. 2002). In diploid cells, drug-resistant isolates are, for the most part, homozygous for the genetic mutations selected by drug-related external pressures (Rustad *et al*. 2002). This is evidenced by the observation that loss of heterozygosity (LOH) in a series of clinical isolates led to selection for an altered ‘fluconazole-resistant’ allele that enhanced antifungal resistance (Rustad *et al*. 2002). Fluconazole and other stresses intensify the frequency with which these genomic mutations occur (Rustad *et al*. 2002; Forche *et al*. 2011; Harrison *et al*. 2014). Much of our understanding regarding drug adaptation and mating comes from the *C. albicans* literature. *Candidaglabrata* is currently assumed to be asexual (Boisnard *et al*. 2015), and there is little information about how *S. cerevisiae* sexual reproduction impacts antifungal adaptation.

Typically, heterozygosity of the *MTL* locus in *C. albicans* hinders cells from mating (Rustad *et al*. 2002; Popp *et al*. 2019). However, genome rearrangements, including transient aneuploidies, mitotic recombination and whole-chromosome loss or duplication can result in *MTL* homozygosity which, in turn, allows for mating-competency to be achieved (Popp *et al*. 2019). *MTL* homozygosity is not sufficient to confer fluconazole drug resistance, but homozygosity of other genes, such as *ERG11* and drug efflux pumps, play an important role in this process (Rustad *et al*. 2002; Pujol *et al*. 2003; Popp *et al*. 2019). Mating in *C. albicans* populations usually occurs between cells within a clonal population, which can be used by cells as a mechanism to combine advantageous traits for adaptation and resistance to antifungal drugs. Fluconazole-induced *MTL* homozygous cells can also become homozygous for antifungal resistance mutations (Popp *et al*. 2019). Popp *et al*. (2019) observed that fluconazole-induced *MTL* homozygous progeny were mating competent, but the initial mating product of these parental strains did not exhibit higher drug resistance than parent cells until exposed to additional selective pressure. These findings suggest that fluconazole treatment selects for resistance mutations and promotes genomic alterations that confer mating competence, which can propagate mutations linked with fluconazole resistance (Popp *et al*. 2019).

Azole resistance has also been linked with aneuploidy, which can improve stress resistance by increased gene dosage for key adaptive mechanisms. Azole-resistant aneuploids can be derived from *C. albicans* tetraploids which are formed *in vitro* by fluconazole-induced mitotic collapse (Harrison *et al*. 2014). In addition, an isochromosome formed by a specific segmental aneuploidy of the two left arms of chromosome 5 (Ch5) in *C. albicans* confers azole resistance (Selmecki, Forche and Berman 2006). This resistance strategy provides additional copies of *ERG11* and *TAC1* which encode the azole-targeted enzyme in the ergosterol biosynthetic pathway and a transcription factor that positively regulates ABC transporters involved in azole efflux, respectively (Selmecki, Forche and Berman 2006). More recent work has discovered that caspofungin can induce LOH and changes in DNA content in both diploids and tetraploids of *C. albicans* (Avramovska and Hickman 2019). Interestingly, *C. albicans* genome instability also can be induced with other cell wall perturbing agents, including calcofluor white (Avramovska and Hickman 2019).

While much of what we know concerning aneuploidy and drug resistance stems from *C. albicans* research, a *C. glabrata* isolate is the first known case of aneuploidy linked with clinical azole resistance (vanden Bossche *et al*. 1992). The chromosome encoding *ERG11* was duplicated in its entirety in this clinical isolate. Further, the use of aneuploidy to overcome stress is not restricted to pathogenic fungi. *Saccharomyces cerevisiae* can employ aneuploidy to cope with nutrient limitation and proteotoxic stresses (Mulla, Zhu and Li 2014). However, aneuploidy is a risky adaptive mechanism that is often associated with fitness defects due to either increased gene dosage or LOH of many genes with potentially deleterious mutations.

### GOF and other mutations

We briefly discussed above how antifungal resistance can be acquired via key GOF mutations. These mutations typically regulate antifungal susceptibility by altering patterns of target gene expression, with targets including efflux pumps, drug targets or transcriptional regulators of efflux pumps and lipid biosynthesis (such as Ca*TAC1*, Sc/Cg*PDR1*, Ca*MRR1 and* Sc/Cg/Ca*UPC2*; Dunkel *et al*. 2008b; Morschhauser *et al*. 2007; Lohberger, Coste and Sanglard 2014). Although GOF mutations can be beneficial to cell survival during antifungal exposure, these mutations can potentially affect fungal virulence and fitness in the absence of selective drug pressure.

GOF mutation fitness has been investigated both *in vitro* and *in vivo* for *C. albicans*. Strains carrying hyperactive alleles of *TAC1* (N9777D), *MRR1* (G963S) and *UPC2* (G648D), which confer azole resistance, were assessed for virulence in a systemic murine infection model (Lohberger, Coste and Sanglard 2014). Lohberger, Coste and Sanglard (2014) showed that *TAC1* and *MRR1* GOF mutations did not significantly affect *C. albicans* virulence compared to wild-type. However, *UPC2* GOF led to a significant decrease in virulence and reduced kidney fungal burden when compared to the wild-type strain (Lohberger, Coste and Sanglard 2014). Additionally, *UPC2* GOF mutations also delayed *C. albicans* filamentation upon phagocytosis by murine macrophages, which may partly explain the virulence defects associated with this mutation *in vivo* (Lohberger, Coste and Sanglard 2014). Interestingly, a strain combining *UPC2* GOF alleles with the GOF mutation in *MRR1* did not rescue the *UPC2* virulence defect, but rather attenuated virulence further (Lohberger, Coste and Sanglard 2014). Given that azole resistance related to *UPC2/ERG11* overexpression is a common problem in the clinic it is possible that cells can compensate for the negative fitness effect of this GOF to thrive under host-imposed conditions (Flowers *et al*. 2012; Lohberger, Coste and Sanglard 2014).

*FKS* mutations at two ‘hot spots’ are a major fungal solution for generating echinocandin resistance. For example, *FKS2* T1987C enhances *C. glabrata* echinocandin resistance, but at the expense of *in vitro* fitness (Singh-Babak *et al*. 2012). Cells harboring this allele had a growth defect compared to wild-type in the absence of selection. However, this defect could be compensated by a GOF mutation to *CDC55* (C463T), which is one of the few characterized compensatory mutations for rescuing fitness in antifungal resistant isolates (Singh-Babak *et al*. 2012). *Saccharomycescerevisiae* has been used as a model system to investigate acquired resistance via *FKS* mutations identified in echinocandin-resistant *Candida* and other fungal species (Johnson, Katiyar and Edlind 2011). This model has successfully replicated echinocandin resistance driven by mutations observed in *Candida parapsilosis* and *Fusarium solani FKS* genes. Whether this model could be used to help identify adjuvant compounds to improve echinocandin efficacy remains to be seen.

*Candida* *glabrata* has an additional mechanism for rapidly generating potentially advantageous mutations during drug treatment that involves altering the mismatch repair and double-strand break pathways. Mutations in *MSH2*, a gene involved in mismatch repair, were identified in ∼55% of *C. glabrata* clinical isolates (Healey *et al*. 2016). These mutations conferred a hyper-mutable phenotype resulting in elevated resistance to azoles and echinocandins *in vitro. MSH2* deletion increased echinocandin resistance *in vivo*, though this *C. glabrata* strain was partially outcompeted by wild-type in a mixed inocula murine gastrointestinal colonization model (Healey *et al*. 2016). Mutations in mismatch repair and double-strand DNA break repair genes in *C. albicans* also give rise to drug resistance more rapidly than wild-type cells (Legrand *et al*. 2007).

## TRANSCRIPTOMICS, PROTEOMICS AND METABOLOMICS INSIGHTS INTO ANTIFUNGAL ADAPTATION

Is antifungal resistance a feature of phenotypic heterogeneity within populations, is it adaptation to specific drug insults or is it a combination of these processes? To address this question, ‘omics studies have explored timed responses of drug-susceptible and drug-resistant populations to antifungals (Tables 2–4). While we found many studies that investigated adaptation using qRT-PCR and other targeted analyses, this section will discuss only ‘omics-driven research into antifungal adaptation.

**Table 2.** List of *S. cerevisiae* ‘omics datasets with a brief description of methodology. A total of two studies include *C. glabrata* datasets. (Abbreviations: 5-FC, 5-flucytosine; FCZ, fluconazole; MCN, miconazole; CTZ, clotrimazole; KCZ, ketoconazole; ICZ, itraconazole; CSP, caspofungin and AmB, amphotericin B.)

Citation	Species	Analysis	Strain	Drug	Methods details
Zhang *et al*. (2002)	*S. cerevisiae*	Transcriptomics (Microarray)	L1190	5-FC, 25 µg/mL	OD_600_ ∼0.8, 30°C YPD, exposed to 5-FC for 90 min, *n* = 1
				(0.5x MIC_100_)	
Agarwal *et al*. (2003)	*S. cerevisiae*	Transcriptomics (Microarray)	S288c	AmB, 0.12 µg/mL	OD_600_ ∼0.2, 30°C, SD, exposed to drug for 3 h, *n* = 2
				5-FC, 0.3 µg/mL	
				CSP, 0.02 µg/mL	
				KCZ, 56 µg/mL	
Reinoso-Martin *et al*. (2003)	*S. cerevisiae*	Transcriptomics (Microarray)	BY4741	CSP, 10 ng/mL	OD_600_ ∼1, 30°C YPD, exposed to CSP for 1, 2 and 3 h, *n* = 4
Kuo *et al*. (2010)	*S. cerevisiae*,	Transcriptomics (Microarray)	BY4741	FCZ, 4 µg/mL (MIC_50_)	OD_600_ ∼0.05–0.2, 30°C YPD, cells
	*C. glabrata*		CBS138		harvested 0, 1/3, 2/3, 1, 2 or 4 doubling times, *n* = 3
Nishikawa *et al*. (2016)	*S. cerevisiae*,	Transcriptomics (RNA-Seq)	BY4741	KCZ, 40 µM	OD_600_ ∼0.8, 30°C YPD, treated with DMSO 8 h, then KCZ for 15 min; *n* = 3
	*C. glabrata*		DSY562		
Pang *et al*. (2017)	*S. cerevisiae*	Transcriptomics (RNA-Seq)	S288c	AmB, 0.03 µg/mL	30°C RPMI-1640, 50–60 min drug treatment; *n* = 3
Garcia *et al*. (2017)	*S. cerevisiae*	Transcriptomics (Microarray)	BY4741	CSP, 15 ng/mL	OD_600_ ∼0.2, 30°C YPD, 2 h drug treatment; *n* = 3
ATripathi *et al*. (2020)	*S. cerevisiae*	Transcriptomics (RNA-Seq)	S288c	CSP, 0.03 µg/mL	OD_600_ ∼0.1, 30°C SD +/− drug for ∼4 h, *n* = 3
Messner *et al*. (2021)	*S. cerevisiae*	Proteomics (ScanningSWATH)	BY4741	MCN, KCZ, ICZ and CTZ, 10 µM	Overnight 30°C SD transferred to 96-well plate, exposed to drug overnight; *n* = 3–4

**Table 3.** List of *C. glabrata* antifungal ‘omics datasets with a brief description of methodology. (Abbreviations: 5-FC, 5-flucytosine; FCZ, fluconazole; CTZ, clotrimazole; KCZ, ketoconazole and AmB, amphotericin B.)

Citation	Species	Analysis	Strain	Drug	Methods details
Caudle (unpublished data)	*C. glabrata*	Transcriptomics (Microarray)	200989	FCZ, 64 µg/mL	OD_600_ ∼0.2, 30°C YPD, +/− drug 2.5 h, *n* = 3
				2x MIC	
Kuo *et al*. (2010)	*S. cerevisiae*,	Transcriptomics (Microarray)	BY4741	FCZ, 4 µg/mL	OD_600_ ∼0.05–0.2, 30°C YPD, cells
	*C. glabrata*		CBS138	MIC_50_	Harvested 0, 1/3, 2/3, 1, 2 or 4 doubling times, *n* = 3 (2x technical)
Nishikawa *et al*. (2016)	*S. cerevisiae*,	Transcriptomics (RNA-Seq)	BY4741	KCZ, 40 µM	OD_600_ ∼0.8, 30°C YPD, treated with DMSO 8 h, then KCZ for 15 min; *n* = 3
	*C. glabrata*		DSY562		
Pais *et al*. (2020)	*C. glabrata*	Transcriptomics (RNA-Seq)	KUE100	FCZ, 150 µg/mL	30°C basal medium to mid-exponential phase, treated +/− drug 1 h, *n* = 3 (2x technical)
Alves *et al*. (2020)	*C. glabrata*	Transcriptomics (RNA-Seq)	CBS138	FCZ, 1250 µg/mL	Pre-formed biofilms +/− drug at 37°C in RPMI pH 7.0, 24 h, *n* = 3
Pais *et al*. (2016a)	*C. glabrata*	Proteomics (iTRAQ-MS)	66032	5-FC, 4 µg/mL	30ºC Basal medium, +/− 5-FC 1 h, *n* = 3
Pais *et al*. (2016b)	*C. glabrata*	Proteomics (iTRAQ-MS)	66032	CTZ, 100 µg/mL	OD_600_ ∼0.4, 30°C basal medium, 1 h, *n* = 3

**Table 4.** List of *C. albicans* ‘omics datasets with a brief description of methodology. (Abbreviations: FCZ, fluconazole; MCN, miconazole; KCZ, ketoconazole; CSP, caspofungin and AmB, amphotericin B.)

Citation	Species	Analysis	Strain	Drug	Methods details
Liu *et al*. (2005)	*C. albicans*	Transcriptomics (Microarray)	SC5314	KCZ, 19.13 µg/mL	OD_600_ ∼0.2, 30°C SD, 3 h, *n* = 3
				AmB, 0.029 µg/mL	
				CSP, 0.0075 µg/mL	
				5-FC, 0.098 µg/mL	
Vasicek *et al*. (2014)	*C. albicans*	Transcriptomics (Microarray)	SC5314	FCZ, 10 µg/mL	OD_600_ ∼0.05, 30°C YPD, 6 h, *n* = 2
Keller *et al*. (2015)	*C. albicans*	Transcriptomics (Microarray)	SC5314	FCZ, ∼0.5 µg/mL (IC_50_)	OD_600_ ∼0.4, 30°C RPMI + 10% fetal calf serum, 3 h, *n* = 3
de Cremer *et al*. (2016)	*C. albicans*	Transcriptomics (RNA-Seq)	SC5314	MCN, 75 µM	Pre-formed biofilms +/− drug at 37°C in RPMI, 4 + 24 h, *n* = 3
Shivarathri *et al*. (2019)	*C. albicans*	Transcriptomics (RNA-Seq)	SC5314	CSP, 10 ng/mL	30°C YPD, 15 + 45 min, *n* = 3
Kuloyo *et al*. (2020)	*C. albicans*	Transcriptomics (RNA-Seq)	SC5314	FCZ, 1 µg/mL	RPMI 37°C, adhered to polystyrene 90 min, +/− drug 6 h, *n* = 3
Hoehamer *et al*. (2010)	*C. albicans*	Proteomics (MALDI-ToF)	SC5314	KCZ, 19.13 µg/mL	OD_600_ ∼0.2, 30°C SD, 6 h, *n* = 3
				AmB, 0.029 µg/mL	
				CSP, 0.0075 µg/mL	
Sorgo *et al*. (2011)	*C. albicans*	Proteomics (LC-ESI-MS/MS)	SC5314	FCZ, 0.5 µg/mL	OD_600_ ∼0.05, 37°C YNB-S, 18 h, *n* = 5
Ene *et al*. (2012)	*C. albicans*	Proteomics (LC-MS/MS)	RM1000	Ambisome, 10 µg/mL	OD_600_ ∼0.1, YNB + 2% glucose or lactate, 1 h, *n* = 3
				CSP, 0.08 µg/mL	
				MCN, 25 µg/mL	
Katragkou *et al*. (2016)	*C. albicans*	Metabolomics (GC-MS, UHPLC-Q-TOF/MS and HILIC-QQQ/MS)	SC5314	KCZ, 16 µg/mL	FCZ sensitive and resistant (64 µg/mL) strains, 30°C YPD to 10^8^ cells/mL, *n* = 6

### Azoles

Perhaps unsurprisingly, the majority of the antifungal ‘omics studies that we identified for *S. cerevisiae*, *C. glabrata* and *C. albicans* characterized responses to azoles (i.e. fluconazole, clotrimazole, ketoconazole, miconazole, itraconazole and voriconazole). We submitted these datasets to the *Saccharomyces* Genome Database and *Candida* Genome Database GO Slim Mappers (Cherry *et al*. 2012; Skrzypek *et al*. 2017) to identify the top biological processes that were differentially regulated during drug treatment (Fig. 2A).

A total of two studies were of particular interest because they simultaneously analysed transcriptional responses for *S. cerevisiae* and *C. glabrata* to fluconazole or ketoconazole, respectively (Kuo *et al*. 2010; Nishikawa *et al*. 2016). Both fluconazole and ketoconazole induced significant changes in gene expression associated with lipid and carbohydrate metabolism, induction of transmembrane transporters such as drug transporters and down-regulation of genes involved in rRNA processing or ribosome biogenesis (Kuo *et al*. 2010; Nishikawa *et al*. 2016). These categorical changes in gene expression were also common features in other azole datasets for *C. glabrata* (Caudle 2010; Pais *et al*. 2020), *C. albicans* (Liu *et al*. 2005; Vasicek *et al*. 2014; Weil *et al*. 2017) and for both *C. glabrata* and *C. albicans* grown under biofilm-forming conditions (Alves *et al*. 2020; Kuloyo *et al*. 2020). All three species down-regulated gene expression associated with DNA replication during fluconazole treatment in multiple datasets (Kuo *et al*. 2010; Alves *et al*. 2020; Kuloyo *et al*. 2020), which correlates well with *in vitro* data demonstrating slower growth rates during drug-induced stress (Rosenberg *et al*. 2018). These changes in gene expression largely match our expectations for adaptation to azoles, which would involve alterations in lipid metabolism to remedy the lack of membrane sterols or build-up of toxic intermediates and an attempt to increase membrane transporters to eliminate antifungals from the cytoplasm.

The consistency between these studies is even more remarkable because of the different approaches used: Caudle's study used a clinical isolate of *C. glabrata*, Weil *et al*. (2017) investigated *C. albicans* strains with mistranslation mutations that affected azole resistance and most studies used different concentrations of drug, growth media or time points for analysis. However, looking more globally at the differentially expressed gene datasets, there were some key differences between studies. For example, Kuloyo *et al*. (2020) observed that *C. albicans* biofilms treated with fluconazole down-regulated genes involved in filamentous growth, but Vasicek *et al*. (2014) and Liu *et al*. (2005) observed induction of filamentous growth genes for fluconazole or ketoconazoletreated planktonic cells, respectively. *Candida glabrata* heme and iron homeostasis were altered in fluconazole-treated cells (Caudle 2010; Pais *et al*. 2020), but these processes did not appear to be significantly impacted in the GO Slim analysis for *S. cerevisiae* and *C. albicans*.

Other ‘omics studies have corroborated key aspects of available transcript profiling data. For example, the mevalonate pathway provides important precursors for ergosterol biosynthesis. Consistent with changes in lipid metabolism, *C. albicans* metabolomics data during fluconazole treatment shows a build-up in mevalonate pathway by-products due to the block in ergosterol synthesis (Katragkou *et al*. 2016). The metabolomics data also indicate that *C. albicans* undergoes major changes in central carbon metabolism and decreases amino acid metabolism, though the significance of these changes is unclear. Proteomics studies have characterized the basal prevalence of cytoplasmic and membrane proteins in azole-resistant and azole-susceptible isolates under the working hypothesis that drug-resistant strains will have enriched expression of drug resistance markers, such as efflux pumps. Consistent with the data obtained from drug stress imposed on sensitive cells, *C. albicans* strains that are resistant to fluconazole had enriched expression of proteins associated with lipid metabolic processes (Hooshdaran *et al*. 2004) and decreased prevalence of proteins involved in DNA repair. *Candida glabrata* fluconazole-resistant isolates were enriched for proteins involved in drug efflux and metabolic processes (Shen *et al*. 2015). Unfortunately, a handful of studies on *C. glabrata* azole-resistant isolates are missing specific gene identifying information, but similarly indicated by biological process data that proteins involved in glucose metabolism and cell wall biogenesis were differentially expressed in azole-resistant strains compared to azole-sensitive cells (Loureiro Y Penha *et al*. 2010; Yoo *et al*. 2012, 2013).

Proteomics work with drug-sensitive strains has focused largely on determining membrane or cell wall changes in protein levels in response to azoles. Membrane proteomics have corroborated transcriptional studies on *C. glabrata* adaptation to azoles. For example, clotrimazole treatment induced drug transporter expression, including Tpo1, Snq2 and Pdr5, and downregulated expression of proteins associated with ribosome biogenesis and oxidative phosphorylation pathways (Pais *et al*. 2016b). *Candida albicans* cell wall proteomics studies demonstrated that fluconazole, miconazole and ketoconazole differentially regulated the expression of several cell wall proteins and virulence factors including adhesins (*ALS3* and *ALS4*), GPI-anchored proteins (*PGA4* and *PGA31*) and secreted aspartyl proteases (*SAP7* and *SAP9*; Sorgo *et al*. 2011; Ene *et al*. 2012). Proteomic data on *C. glabrata* virulence factor expression, such as *EPA* adhesins, in response to azole treatment is lacking. However, transcriptomic data suggests that Cg*EPA1*, a sub-telomerically encoded adhesin that plays an important role in human epithelial cell adhesion, is upregulated in multiple fluconazole-resistant clinical isolates compared to fluconazole-sensitive isolates (Caudle 2010). *CgEPA1* is a homolog of *ScFLO10*, a flocculin important for cell-to-cell adhesion. *ScFLO10* also is upregulated in yeast cells grown in the presence of fluconazole (Kuo *et al*. 2010). Cell-to-cell and cell-to-substrate adhesion are important for biofilm formation, which can modulate antifungal efficacy. It remains to be seen whether azole-induced adhesin expression constitutes a concerted effort to form biofilms as part of a protective adaptive response to azole exposure.

### Echinocandins

Echinocandins are the preferred first line of treatment for invasive candidiasis. However, out of the six studies that used ‘omics techniques to interrogate echinocandin adaptation in fungal cells, none included *C. glabrata*. Further, all of the studies we found focused on characterizing responses to caspofungin but not anidulafungin or micafungin.

In 2003, back-to-back microarray studies in *S. cerevisiae* provided a first glimpse of yeast adaptive responses to caspofungin (Agarwal *et al*. 2003; Reinoso-Martin *et al*. 2003). Unsurprisingly, genes involved in cell wall organization or biogenesis were the most significantly enriched biological process during caspofungin treatment (Agarwal *et al*. 2003; Reinoso-Martin *et al*. 2003). Caspofungin also induced the expression of genes involved in sporulation or ‘response to chemical’ in both datasets (Agarwal *et al*. 2003; Reinoso-Martin *et al*. 2003). In contrast, genes involved in transmembrane or ion transport were down-regulated in response to caspofungin treatment. *In vitro* data, thus far supports the conclusion that echinocandins are not substrates for the ABC transporters that mediate azole efflux in azole-resistant strains (Niimi *et al*. 2006). More recent transcript profiling studies (Garcia *et al*. 2017; Tripathi *et al*. 2020) show consistent changes in gene expression with those described by Agarwal *et al*. (2003). In particular, genes involved in cell wall biogenesis, carbohydrate metabolism and protein phosphorylation/modification were enriched during caspofungin treatment while genes involved in conjugation and ion or transmembrane transport were down-regulated (Agarwal *et al*. 2003; Garcia *et al*. 2017; Tripathi *et al*. 2020). Reinoso-Martin *et al*. (2003) showed enrichment of cell cycle and DNA replication machinery while Agarwal *et al*. (2003) observed enrichment in genes involved in carbohydrate and amino acid metabolism.

Proteomics data from *C. albicans* during caspofungin treatment presents some similarities to the *S. cerevisiae* datasets. In *C. albicans*, proteins involved in carbohydrate metabolism, response to chemical and cell-cycle regulation were enriched during caspofungin exposure in two studies (Liu *et al*. 2005; Hoehamer *et al*. 2010). Shivarathri *et al*. (2019) investigated *C. albicans* responses to caspofungin over 15 and 45 min of exposure. Unique to *C. albicans*, caspofungin treatment differentially regulated filamentous growth gene expression with key genes involved in hyphal growth (*HGC1*, *RFX2* and *UME6*) up-regulated within 45 min of drug exposure (Shivarathri *et al*. 2019). Similar to *S. cerevisiae*, *C. albicans*-induced expression of genes involved in carbohydrate metabolism and response to stress and down-regulated the expression of genes associated with lipid metabolism, protein catabolism and cellular homeostasis (Shivarathri *et al*. 2019).

*In vitro* and *in vivo* data from *C. albicans* paints a striking image of how caspofungin affects cell viability and virulence. Cells starved for cell wall β-glucan due to inhibited synthesis compensate by dramatically increasing chitin content in the inner cell wall (Lee *et al*. 2012; Walker, Gow and Munro 2013). This alteration in inner cell wall composition has consequences for innate immune interactions and virulence. *Candida albicans* cells treated with caspofungin are hypovirulent in mice, but do not appear to be cleared by immune cells and replicate to high fungal burdens in murine kidneys (Lee *et al*. 2012). Interestingly, the compensatory adaptation in chitin synthesis in response to caspfungin is not conserved in *C. glabrata*, though cell wall integrity appears to be important for *in vivo* echinocandin tolerance (Garcia-Rubio *et al*. 2021). In *S. cerevisiae*, a deletion library screen identified 25 mutations that resulted in enhanced caspofungin resistance (Garcia *et al*. 2015). Mutations related to lipid metabolism (C*SG2, ELO2, ELO3, CKA2* and *SUR1*), sterol biosynthesis (*SAY1*, *ERG3* and *NSG2*), fatty acid synthesis (*ETR1*), translocation of phospholipids across the plasma membrane (*LEM3*) and lower glucan synthase activity (*WSC1, ELO2*and*ELO3*) conferred hyper-resistance to caspofungin (Garcia *et al*. 2015). Understanding physical and genetic adaptation mechanisms to echinocandins and their conservation across species could provide useful insights into how to overcome resistance through adjuvant therapy targeted against key adaptive traits, such as other cell wall biogenesis pathways.

### Polyenes

AmB is an effective and robust last line of defense against invasive fungal infections. Given its length of use in the clinic, we were surprised to find few ‘omics studies on AmB responses and adaptation in *S. cerevisiae* or *Candida* species.

A total of two studies, ∼14-years-apart, investigated AmB effects on *S. cerevisiae* transcription using microarray (Agarwal *et al*. 2003) and RNA-Seq (Pang *et al*. 2017) approaches. Both studies have notable consistency in the biological processes enriched by treatment with AmB, which included genes involved in transmembrane and ion transport, cell wall organization, amino acid metabolism and transcription by RNA polymerase II (Agarwal *et al*. 2003; Pang *et al*. 2017). Both studies also noted down-regulation of genes involved in cell-cycle progression. Agarwal *et al*. (2003) observed differential regulation of carbohydrate metabolism and cytoskeletal organization genes while Pang *et al*. (2017) reported changes in gene expression related to lipid metabolic processes, mitochondrion organization, rRNA and tRNA processing and ribosome biogenesis.

In *C. albicans*, AmB adaptation has been investigated using proteomics to determine changes in cell wall and cytoplasmic protein levels (Hoehamer *et al*. 2010; Ene *et al*. 2012). Hoehamer *et al*. (2010) identified several proteins that were enriched during AmB treatment that are consistent with transcriptional changes noted by Agarwal *et al*. (2003) in *S. cerevisiae*. In particular, proteins involved in nucleobase, carbohydrate and amino acid metabolism, transmembrane transport and response to oxidative stress were more prevalent in *C. albicans* cells exposed to AmB compared to untreated cells. Ene *et al*. (2012) characterized changes in cell wall protein expression during AmB treatment and discovered that proteins involved in β-glucan maintenance (Phr2, Crh11 and Eng1) were enriched during drug exposure. Other cell wall proteins were less prevalent during drug exposure, including the chitinase Cht1, secreted aspartyl protease Sap9 and virulence factor Rbt4 (Ene *et al*. 2012).

Polyene perturbations to membrane fluidity and homeostasis clearly have large effects on lipid metabolism, membrane protein incorporation and cell wall organization. Ene *et al*. (2012) also highlights how AmB treatment may have the added benefit of negatively regulating virulence factor expression. While clinical resistance to AmB is rare, further study on the adaptation of fungal pathogens to this drug is warranted especially as the first observed cases of pan-resistant *C. auris* are being reported in the USA (Lyman *et al*. 2021).

### 5-FC

Finally, we identified two *S. cerevisiae* microarray studies, one *C. albicans* microarray study and one *C. glabrata* proteomics study that investigated cell responses to treatment with 5-FC.

*Saccharomyces cerevisiae* microarray investigations in 2002 and 2003 showed that 5-FC treated cells responded to drug insult by up-regulating gene expression associated with DNA replication, DNA repair and cell-cycle machinery (Zhang *et al*. 2002; Agarwal *et al*. 2003). Transmembrane and ion transporters were differentially regulated. Some transport classes, such as Mep ammonium transporters, were down-regulated during drug treatment and other genes, such as antiporter family member *TPO2*, were up-regulated (Agarwal *et al*. 2003). Genes involved in amino acid metabolism and transcription via RNA polymerase II were down-regulated during 5-FC exposure (Zhang *et al*. 2002; Agarwal *et al*. 2003).

The transcript profiling data from *C. albicans* and proteomics data from *C. glabrata* cells treated with 5-FC bear little resemblance to *S. cerevisiae* transcript profiling (Liu *et al*. 2005; Pais *et al*. 2016a). Similar to *S. cerevisiae*, transmembrane transporters were differentially regulated by 5-FC treatment, but the most enriched biological processes in *C. albicans* and *C. glabrata* involved translational regulation and ribosome biogenesis rather than DNA repair. In fungal cells, 5-FC is converted to 5-FU, which is further converted into several metabolites that affect translation and cause DNA damage. What these datasets appear to suggest is that 5-FC treatment differentially affects *Candida* species and *S. cerevisiae* biological responses (Zhang *et al*. 2002; Agarwal *et al*. 2003; Liu *et al*. 2005; Pais *et al*. 2016a). *Candida glabrata* and *C. albicans* appear to be more sensitive to translational inhibition caused via 5-FU incorporation into mRNA, whereas *S. cerevisiae* transcriptional changes indicate sensitivity to the depletion of dTTP via 5-FU inhibition of thymidylate synthase, resulting in dUTP incorporation into DNA and, ultimately, DNA damage. Further transcriptomics and proteomics work are needed to confirm these observations of differing sensitivities to 5-FC in *C. albicans*, *C. glabrata* and *S. cerevisiae*.

## CONCLUSIONS

In this review, we have discussed the resistance mechanisms and ‘omics-determined physiological responses of *S. cerevisiae*, *C. glabrata* and *C. albicans* to the major classes of antifungal drugs used against invasive candidiasis. Some resistance mechanisms and adaptive responses are conserved between these pathogenic and non-pathogenic fungi, particularly against azole treatment, where cells showed adaptation in lipid metabolism and enrichment of efflux pump expression (Fig. 2). Some adaptive mechanisms were less well-conserved, such as the datasets suggesting that *C. albicans* and *C. glabrata* responses to 5-FC were driven more by translational inhibition compared to *S. cerevisiae*, which appeared to preferentially up-regulate genes involved in DNA repair (Fig. 2B and supplemental data). These observations indicate that *S. cerevisiae* may be an excellent model organism for understanding responses to certain antifungals but may be more difficult to extrapolate data for others. Additional investigations on model and non-model organism antifungal responses are needed to address the limitations of basing antifungal response paradigms on data from normally non-pathogenic organisms.

We initially set out to perform a systematic review of ‘omics datasets on antifungal adaptation. However, our efforts to conduct this review systematically were hindered by three fundamental issues. First, sourcing articles using broad keyword search strings, such as the use of ‘’antifungal’ AND ‘transcriptomics’ AND ‘*species’*’, returned fewer than 10% of the relevant articles highlighted in this review. Substituting ‘antifungal’ with a specific antifungal name only modestly improved search success. Second, while there is an abundance of ‘omics literature on azole adaptation, differences in strains, media, growth conditions and timepoints used made data comparisons difficult. We have attempted to address this issue by doing light-touch comparisons of differentially regulated biological processes from each study and highlighting consistencies between studies which we consider even more robust given the technical differences in approaches. Finally, compared to the azole literature there is a relative drought of information for micafungin, anidulafungin, isavuconazole, voriconazole, AmB and 5-FC. What the field needs in the future are large-scale studies covering multiple timepoints, strains and antifungal drugs to help draw more robust conclusions about how antifungals influence fungal adaptation, host interactions and the development of antifungal resistance. Next-generation technologies, such as single-cell RNA sequencing and multi-omics approaches, will be important tools to address the dynamic sub-population changes behind the development of tolerance vs. mutational approaches to surviving antifungals.

Antifungals significantly affect several aspects of fungal physiology including carbon and lipid metabolism, cell wall organization, membrane protein expression, cell division and genomic stability. Each of these processes in turn can affect fungal fitness, host interactions and pathogenesis. As we have discussed earlier in this review, there are several mechanisms that lead to resistance and survival in the face of antifungal insults, though the mechanisms driving tolerance are poorly understood. We are approaching a clinical cliff where the limited repertoire of available antifungals is coming up short against emerging pan-resistant fungal pathogens. We need carefully designed ‘omics and multi-omics studies to better understand how genetic and physiological rewiring events during drug exposure alter antifungal resistance and host interactions to identify new avenues for the development of adjuvant or novel therapeutic strategies.

## Supplementary Material

foab070_Supplemental_Files

## References

[bib2] Allen U . Antifungal agents for the treatment of systemic fungal infections in children. Paediatr Child Health. 2010;15:603–8. 22043144 PMC3009569

[bib1] Agarwal AK , Rogers PD, Baerson SR et al. Genome-wide expression profiling of the response to polyene, pyrimidine, azole, and echinocandin antifungal agents in *Saccharomyces**cerevisiae*. J Biol Chem. 2003;278:34998–5015. 12824174 10.1074/jbc.M306291200

[bib3] Alves R , Kastora SL, Gomes-Goncalves A et al. Transcriptional responses of Candida glabrata biofilm cells to fluconazole are modulated by the carbon source. NPJ Biofilms Microbiomes. 2020;6:4–020-0114-5. 31993211 10.1038/s41522-020-0114-5 PMC6978337

[bib4] Anderson JB , Sirjusingh C, Parsons AB et al. Mode of selection and experimental evolution of antifungal drug resistance in *Saccharomyces**cerevisiae*. Genetics. 2003;163:1287–98. 12702675 10.1093/genetics/163.4.1287 PMC1462505

[bib5] Avramovska O , Hickman MA. The magnitude of *Candida**albicans* stress-induced genome instability results from an interaction between ploidy and antifungal drugs. G3 (Bethesda). 2019;9:4019–27. 31585926 10.1534/g3.119.400752 PMC6893200

[bib6] Bennett JE , Izumikawa K, Marr KA. Mechanism of increased fluconazole resistance in *Candida**glabrata* during prophylaxis. Antimicrob Agents Chemother. 2004;48:1773–7. 15105134 10.1128/AAC.48.5.1773-1777.2004 PMC400565

[bib7] Bhattacharya S , Sae-Tia S, Fries BC. Candidiasis and mechanisms of antifungal resistance. Antibiotics (Basel). 2020;9. DOI: 10.3390/antibiotics9060312. 10.3390/antibiotics9060312 PMC7345657 32526921

[bib8] Boisnard S , Zhou Li Y, Arnaise S et al. Efficient mating-type switching in *Candida glabrata* induces cell death. PLoS ONE. 2015;10:e0140990. 26491872 10.1371/journal.pone.0140990 PMC4619647

[bib9] Bojsen R , Regenberg B, Folkesson A. *Saccharomyces cerevisiae* biofilm tolerance towards systemic antifungals depends on growth phase. BMC Microbiol. 2014;14:305. 25472667 10.1186/s12866-014-0305-4 PMC4258017

[bib10] Bojsen R , Regenberg B, Gresham D et al. A common mechanism involving the TORC1 pathway can lead to amphotericin B-persistence in biofilm and planktonic *Saccharomyces**cerevisiae* populations. Sci Rep. 2016;6:21874. 26903175 10.1038/srep21874 PMC4763212

[bib11] Bongomin F , Gago S, Oladele RO et al. Global and multi-national prevalence of fungal diseases—estimate precision. J Fungi (Basel). 2017;3:57. 10.3390/jof3040057 PMC5753159 29371573

[bib12] Brun S , Bergès T, Poupard P et al. Mechanisms of azole resistance in petite mutants of *Candida**glabrata*. Antimicrob Agents Chemother. 2004;48:1788–96. 15105136 10.1128/AAC.48.5.1788-1796.2004 PMC400549

[bib13] Caplan T , Polvi EJ, Xie JL et al. Functional genomic screening reveals core modulators of echinocandin stress responses in *Candida**albicans*. Cell Rep. 2018;23:2292–8. 29791841 10.1016/j.celrep.2018.04.084

[bib14] Castanheira M , Woosley LN, Diekema DJ et al. Low prevalence of *fks1* hot spot 1 mutations in a worldwide collection of *Candida* strains. Antimicrob Agents Chemother. 2010;54:2655–9. 20368396 10.1128/AAC.01711-09 PMC2876398

[bib15] Caudle KE . Transcriptional regulation of azole antifungal resistance and tolerance in *Candida glabrata*. *Ph.D. Thesis*. University of Tennessee Health Science Center, Theses and Dissertations (ETD) 2010.

[bib16] CDC . Invasive Candidiasis Statistics, https://www.cdc.gov/fungal/diseases/candidiasis/invasive/statistics.html 2021;2021.

[bib17] Chandra J , Mukherjee PK, Leidich SD et al. Antifungal resistance of *Candida*l biofilms formed on denture acrylic in vitro. J Dent Res. 2001;80:903–8. 11379893 10.1177/00220345010800031101

[bib18] Cherry JM , Hong EL, Amundsen C et al. *Saccharomyces* genome database: the genomics resource of budding yeast. Nucleic Acids Res. 2012;40:D700–5. 22110037 10.1093/nar/gkr1029 PMC3245034

[bib19] Coste AT , Karababa M, Ischer F et al. TAC1, transcriptional activator of CDR genes, is a new transcription factor involved in the regulation of *Candida albicans* ABC transporters CDR1 and CDR2. Eukaryot Cell. 2004;3:1639–52. 15590837 10.1128/EC.3.6.1639-1652.2004 PMC539021

[bib20] Cota JM , Grabinski JL, Talbert RL et al. Increases in *SLT2* expression and chitin content are associated with incomplete killing of *Candida**glabrata* by caspofungin. Antimicrob Agents Chemother. 2008;52:1144–6. 18086838 10.1128/AAC.01542-07 PMC2258485

[bib21] Cowen LE , Sanglard D, Howard SJ et al. Mechanisms of antifungal drug resistance. Cold Spring Harb Perspect Med. 2014;5:a019752. 25384768 10.1101/cshperspect.a019752 PMC4484955

[bib139_739_072822] De Cremer K, De Brucker K, Staes I et al. Stimulation of superoxide production increases fungicidal action of miconazole against Candida albicans biofilms. Sci Rep. 2016;6:27463. 27272719 10.1038/srep27463 PMC4895440

[bib22] Demuyser L , Swinnen E, Fiori A et al. Mitochondrial cochaperone mge1 is involved in regulating susceptibility to fluconazole in *Saccharomyces**cerevisiae* and *Candida* species. mBio. 2017;8:e00201–17. 28720726 10.1128/mBio.00201-17 PMC5516249

[bib23] Diekema D , Arbefeville S, Boyken L et al. The changing epidemiology of healthcare-associated candidemia over three decades. Diagn Microbiol Infect Dis. 2012;73:45–8. 22578938 10.1016/j.diagmicrobio.2012.02.001

[bib24] Dodgson AR , Dodgson KJ, Pujol C et al. Clade-specific flucytosine resistance is due to a single nucleotide change in the *FUR1* gene of *Candida**albicans*. Antimicrobial Agents Chemother. 2004;48:2223–7. 10.1128/AAC.48.6.2223-2227.2004 PMC415630 15155225

[bib25] Douglas CM , D'Ippolito JA, Shei GJ et al. Identification of the *FKS1* gene of *Candida**albicans* as the essential target of 1,3-beta-D-glucan synthase inhibitors. Antimicrob Agents Chemother. 1997;41:2471–9. 9371352 10.1128/aac.41.11.2471 PMC164147

[bib26] Dunkel N , Blass J, Rogers PD et al. Mutations in the multi-drug resistance regulator *MRR1*, followed by loss of heterozygosity, are the main cause of *MDR1* overexpression in fluconazole-resistant *Candida**albicans* strains. Mol Microbiol. 2008a;69:827–40. 18577180 10.1111/j.1365-2958.2008.06309.x PMC2678921

[bib27] Dunkel N , Liu TT, Barker KS et al. A gain-of-function mutation in the transcription factor upc2p causes upregulation of ergosterol biosynthesis genes and increased fluconazole resistance in a clinical *Candida**albicans* isolate. Eukaryot Cell. 2008b;7:1180–90. 18487346 10.1128/EC.00103-08 PMC2446669

[bib29] Edlind TD , Katiyar SK. Mutational analysis of flucytosine resistance in *Candida**glabrata*. Antimicrob Agents Chemother. 2010;54:4733–8. 20823283 10.1128/AAC.00605-10 PMC2976130

[bib30] Ene IV , Heilmann CJ, Sorgo AG et al. Carbon source-induced reprogramming of the cell wall proteome and secretome modulates the adherence and drug resistance of the fungal pathogen *Candida albicans*. Proteomics. 2012;12:3164–79. 22997008 10.1002/pmic.201200228 PMC3569869

[bib31] Erbs P , Exinger F, Jund R. Characterization of the *Saccharomyces**cerevisiae FCY1* gene encoding cytosine deaminase and its homologue *FCA1* of *Candida**albicans*. Curr Genet. 1997;31:1–6. 9000374 10.1007/s002940050169

[bib32] Ferrari S , Ischer F, Calabrese D et al. Gain of function mutations in cg*pdr1* of *Candida**glabrata* not only mediate antifungal resistance but also enhance virulence. PLoS Pathog. 2009;5:e1000268. 19148266 10.1371/journal.ppat.1000268 PMC2607542

[bib33] Flowers SA , Barker KS, Berkow EL et al. Gain-of-function mutations in *UPC2* are a frequent cause of *ERG11* upregulation in azole-resistant clinical isolates of *Candida**albicans*. Eukaryot Cell. 2012;11:1289–99. 22923048 10.1128/EC.00215-12 PMC3485914

[bib34] Forche A , Abbey D, Pisithkul T et al. Stress alters rates and types of loss of heterozygosity in *Candida**albicans*. mBio. 2011;2. DOI: 10.1128/mBio.00129-11. 10.1128/mBio.00129-11 PMC3143845 21791579

[bib36] Garcia R , Botet J, Rodriguez-Pena JM et al. Genomic profiling of fungal cell wall-interfering compounds: identification of a common gene signature. BMC Genomics. 2015;16:683. 26341223 10.1186/s12864-015-1879-4 PMC4560923

[bib35] Garcia R , Bravo E, Diez-Muniz S et al. A novel connection between the cell wall integrity and the PKA pathways regulates cell wall stress response in yeast. Sci Rep. 2017;7:5703. 28720901 10.1038/s41598-017-06001-9 PMC5515849

[bib37] Garcia-Rubio R , Hernandez RY, Clear A et al. Critical assessment of cell wall integrity factors contributing to in vivo echinocandin tolerance and resistance in *Candida**glabrata*. Front Microbiol. 2021;12:702779. 34305871 10.3389/fmicb.2021.702779 PMC8298035

[bib38] Geber A , Hitchcock CA, Swartz JE et al. Deletion of the *Candida**glabrata ERG3* and *ERG11* genes: effect on cell viability, cell growth, sterol composition, and antifungal susceptibility. Antimicrob Agents Chemother. 1995;39:2708–17. 8593007 10.1128/aac.39.12.2708 PMC163017

[bib39] Guinea J . Global trends in the distribution of *Candida* species causing candidemia. Clin Microbiol Infect. 2014;20 Suppl 6:5–10. 10.1111/1469-0691.12539 24506442

[bib40] Harrison BD , Hashemi J, Bibi M et al. A tetraploid intermediate precedes aneuploid formation in yeasts exposed to fluconazole. PLoS Biol. 2014;12:e1001815. 24642609 10.1371/journal.pbio.1001815 PMC3958355

[bib42] Healey KR , Challa KK, Edlind TD et al. Sphingolipids mediate differential echinocandin susceptibility in *Candida**albicans* and *Aspergillus**nidulans*. Antimicrob Agents Chemother. 2015;59:3377–84. 25824222 10.1128/AAC.04667-14 PMC4432167

[bib43] Healey KR , Katiyar SK, Raj S et al. CRS-MIS in *Candida**glabrata*: sphingolipids modulate echinocandin-Fks interaction. Mol Microbiol. 2012;86:303–13. 22909030 10.1111/j.1365-2958.2012.08194.x PMC3472958

[bib41] Healey KR , Perlin DS. Fungal resistance to echinocandins and the MDR phenomenon in *Candida* glabrata. J Fungi (Basel). 2018;4. DOI: 10.3390/jof4030105. 10.3390/jof4030105 PMC6162769 30200517

[bib44] Healey KR , Zhao Y, Perez WB et al. Prevalent mutator genotype identified in fungal pathogen *Candida**glabrata* promotes multi-drug resistance. Nat Commun. 2016;7:11128. 27020939 10.1038/ncomms11128 PMC5603725

[bib45] Hoehamer CF , Cummings ED, Hilliard GM et al. Changes in the proteome of *Candida**albicans* in response to azole, polyene, and echinocandin antifungal agents. Antimicrob Agents Chemother. 2010;54:1655–64. 20145080 10.1128/AAC.00756-09 PMC2863685

[bib46] Hooshdaran MZ , Barker KS, Hilliard GM et al. Proteomic analysis of azole resistance in *Candida**albicans* clinical isolates. Antimicrob Agents Chemother. 2004;48:2733–5. 15215138 10.1128/AAC.48.7.2733-2735.2004 PMC434221

[bib47] Hope WW , Tabernero L, Denning DW et al. Molecular mechanisms of primary resistance to flucytosine in *Candida**albicans*. Antimicrob Agents Chemother. 2004;48:4377–86. 15504867 10.1128/AAC.48.11.4377-4386.2004 PMC525410

[bib49] Hull CM , Bader O, Parker JE et al. Two clinical isolates of *Candida**glabrata* exhibiting reduced sensitivity to amphotericin b both harbor mutations in *ERG2*. Antimicrob Agents Chemother. 2012b;56:6417–21. 23027188 10.1128/AAC.01145-12 PMC3497184

[bib48] Hull CM , Parker JE, Bader O et al. Facultative sterol uptake in an ergosterol-deficient clinical isolate of *Candida**glabrata* harboring a missense mutation in *ERG11* and exhibiting cross-resistance to azoles and amphotericin B. Antimicrob Agents Chemother. 2012a;56:4223–32. 22615281 10.1128/AAC.06253-11 PMC3421581

[bib50] Johnson ME , Katiyar SK, Edlind TD. New Fks hot spot for acquired echinocandin resistance in *Saccharomyces**cerevisiae* and its contribution to intrinsic resistance of *scedosporium* species▿. Antimicrob Agents Chemother. 2011;55:3774–81. 21576441 10.1128/AAC.01811-10 PMC3147641

[bib51] Kanafani ZA , Perfect JR. Antimicrobial resistance: resistance to antifungal agents: mechanisms and clinical impact. Clin Infect Dis. 2008;46:120–8. 18171227 10.1086/524071

[bib52] Katragkou A , Alexander EL, Eoh H et al. Effects of fluconazole on the metabolomic profile of *Candida**albicans*. J Antimicrob Chemother. 2016;71:635–40. 26668236 10.1093/jac/dkv381

[bib138_958_072722] Keller P, Müller C, Engelhardt I et al. An Antifungal Benzimidazole Derivative Inhibits Ergosterol Biosynthesis and Reveals Novel Sterols. Antimicrob Agents Chemother. 2015;59:6296–307. 26248360 10.1128/AAC.00640-15 PMC4576113

[bib53] Kelly SL , Lamb DC, Kelly DE et al. Resistance to fluconazole and amphotericin in *Candida**albicans* from AIDS patients. Lancet. 1996;348:1523–4. 10.1016/S0140-6736(05)65949-1 8942815

[bib54] Kodedová M , Sychrová H. Changes in the Sterol Composition of the Plasma Membrane Affect Membrane Potential, Salt Tolerance and the Activity of Multidrug Resistance Pumps in *Saccharomyces cerevisiae*. PLoS ONE. 2015;10:e0139306. 26418026 10.1371/journal.pone.0139306 PMC4587746

[bib55] Kong Y , Wang Q, Cao F et al. *BSC2* enhances cell resistance to AmB by inhibiting oxidative damage in *Saccharomyces**cerevisiae*. Free Radic Res. 2020;54:231–43. 32295440 10.1080/10715762.2020.1751151

[bib56] Kontoyiannis DP , Sagar N, Hirschi KD. Overexpression of erg11p by the regulatable *GAL1* promoter confers fluconazole resistance in *Saccharomyces**cerevisiae*. Antimicrob Agents Chemother. 1999;43:2798–800. 10543768 10.1128/aac.43.11.2798 PMC89564

[bib57] Kuloyo O , Fourie R, Cason E et al. Transcriptome analyses of *Candida**albicans* biofilms, exposed to arachidonic acid and fluconazole, indicates potential drug targets. G3 (Bethesda). 2020;10:3099–108. 32631950 10.1534/g3.120.401340 PMC7466979

[bib58] Kuo D , Tan K, Zinman G et al. Evolutionary divergence in the fungal response to fluconazole revealed by soft clustering. Genome Biol. 2010;11:R77. 20653936 10.1186/gb-2010-11-7-r77 PMC2926788

[bib59] Lamoth F , Lockhart SR, Berkow EL et al. Changes in the epidemiological landscape of invasive candidiasis. J Antimicrob Chemother. 2018;73:i4–i13. 29304207 10.1093/jac/dkx444 PMC11931512

[bib60] Leach MD , Klipp E, Cowen LE et al. Fungal hsp90: a biological transistor that tunes cellular outputs to thermal inputs. Nat Rev Microbiol. 2012;10:693–704. 22976491 10.1038/nrmicro2875 PMC3660702

[bib61] Lee KK , Maccallum DM, Jacobsen MD et al. Elevated cell wall chitin in *Candida**albicans* confers echinocandin resistance in vivo. Antimicrob Agents Chemother. 2012;56:208–17. 21986821 10.1128/AAC.00683-11 PMC3256049

[bib62] Legrand M , Chan CL, Jauert PA et al. Role of DNA mismatch repair and double-strand break repair in genome stability and antifungal drug resistance in *Candida**albicans*. Eukaryot Cell. 2007;6:2194–205. 17965250 10.1128/EC.00299-07 PMC2168241

[bib63] Liu TT , Lee RE, Barker KS et al. Genome-wide expression profiling of the response to azole, polyene, echinocandin, and pyrimidine antifungal agents in *Candida**albicans*. Antimicrob Agents Chemother. 2005;49:2226–36. 15917516 10.1128/AAC.49.6.2226-2236.2005 PMC1140538

[bib64] Lohberger A , Coste AT, Sanglard D. Distinct roles of *Candida**albicans* drug resistance transcription factors *TAC1*, *MRR1*, and *UPC2* in virulence. Eukaryot Cell. 2014;13:127–42. 24243794 10.1128/EC.00245-13 PMC3910953

[bib65] Loureiro Y Penha CV , Kubitschek PH, Larcher G et al. Proteomic analysis of cytosolic proteins associated with petite mutations in *Candida**glabrata*. Braz J Med Biol Res. 2010;43:1203–14. 21085892 10.1590/s0100-879x2010007500125

[bib66] Lyman M , Forsberg K, Dang T et al. Notes from the field: transmission of pan-resistant and echinocandin-resistant *Candida**auris* in health care facilities - Texas and the District of Columbia, January-April 2021. MMWR Morb Mortal Wkly Rep. 2021;70:1022–3. 34292928 10.15585/mmwr.mm7029a2 PMC8297693

[bib67] Mahé Y , Parle-McDermott A, Nourani A et al. The ATP-binding cassette multidrug transporter SNQ2 of *Saccharomyces**cerevisiae*: a novel target for the transcription factors Pdr1 and Pdr3. Mol Microbiol. 1996;20:109–17. 8861209 10.1111/j.1365-2958.1996.tb02493.x

[bib68] Marichal P , Koymans L, Willemsens S et al. Contribution of mutations in the cytochrome P450 14alpha-demethylase (Erg11p, cyp51p) to azole resistance in *Candida**albicans*. Microbiology (Reading). 1999;145:2701–13. 10537192 10.1099/00221287-145-10-2701

[bib69] Markovich S , Yekutiel A, Shalit I et al. Genomic approach to identification of mutations affecting caspofungin susceptibility in *Saccharomyces**cerevisiae*. Antimicrob Agents Chemother. 2004;48:3871–6. 15388447 10.1128/AAC.48.10.3871-3876.2004 PMC521896

[bib70] Martel C , Parker JE, Bader O et al. A clinical isolate of *Candida**albicans* with mutations in *ERG11* (encoding sterol 14alpha-demethylase) and *ERG5* (encoding C22 desaturase) is cross resistant to azoles and amphotericin B. Antimicrob Agents Chemother. 2010;54:3578–83. 20547793 10.1128/AAC.00303-10 PMC2934972

[bib71] Martel CM , Parker JE, Bader O et al. Identification and characterization of four azole-resistant *erg3* mutants of *Candida**albicans*. Antimicrob Agents Chemother. 2010;54:4527–33. 20733039 10.1128/AAC.00348-10 PMC2976150

[bib137_293_072522] Messner CB, Demichev V, Bloomfield N et al. Ultra-fast proteomics with Scanning SWATH. Nat Biotechnol. 2021;39:846–54. 33767396 10.1038/s41587-021-00860-4 PMC7611254

[bib72] Morschhauser J , Barker KS, Liu TT et al. The transcription factor mrr1p controls expression of the *MDR1* efflux pump and mediates multidrug resistance in *Candida**albicans*. PLoS Pathog. 2007;3:e164. 17983269 10.1371/journal.ppat.0030164 PMC2048531

[bib73] Mukherjee PK , Chandra J, Kuhn DM et al. Mechanism of fluconazole resistance in *Candida**albicans* biofilms: phase-specific role of efflux pumps and membrane sterols. Infect Immun. 2003;71:4333–40. 12874310 10.1128/IAI.71.8.4333-4340.2003 PMC165995

[bib74] Mulla W , Zhu J, Li R. Yeast: a simple model system to study complex phenomena of aneuploidy. FEMS Microbiol Rev. 2014;38:201–12. 24118136 10.1111/1574-6976.12048 PMC3951669

[bib75] Muzafar S , Sharma RD, Shah AH et al. Identification of genomewide alternative splicing events in sequential, isogenic clinical isolates of *Candida**albicans* reveals a novel mechanism of drug resistance and tolerance to cellular stresses. mSphere. 2020;5:e00608–20. 32817456 10.1128/mSphere.00608-20 PMC7426172

[bib76] Niimi K , Maki K, Ikeda F et al. Overexpression of *Candida**albicans CDR1*, *CDR2*, or *MDR1* does not produce significant changes in echinocandin susceptibility. Antimicrob Agents Chemother. 2006;50:1148–55. 16569823 10.1128/AAC.50.4.1148-1155.2006 PMC1426986

[bib77] Nishikawa JL , Boeszoermenyi A, Vale-Silva LA et al. Inhibiting fungal multidrug resistance by disrupting an activator-mediator interaction. Nature. 2016;530:485–9. 26886795 10.1038/nature16963 PMC4860947

[bib78] Owsianik G , Balzi l L, Ghislain M. Control of 26S proteasome expression by transcription factors regulating multidrug resistance in *Saccharomyces**cerevisiae*. Mol Microbiol. 2002;43:1295–308. 11918814 10.1046/j.1365-2958.2002.02823.x

[bib81] Pais P , Califórnia R, Galocha M et al. *Candida glabrata* transcription factor rpn4 mediates fluconazole resistance through regulation of ergosterol biosynthesis and plasma membrane permeability. Antimicrob Agents Chemother. 2020;64:e00554–20. 32571817 10.1128/AAC.00554-20 PMC7449212

[bib80] Pais P , Costa C, Pires C et al. Membrane proteome-wide response to the antifungal drug clotrimazole in *Candida**glabrata*: role of the transcription factor CgPdr1 and the drug:H+ antiporters CgTpo1_1 and CgTpo1_2. Mol Cell Proteomics. 2016b;15:57–72. 26512119 10.1074/mcp.M114.045344 PMC4762512

[bib79] Pais P , Pires C, Costa C et al. Membrane proteomics analysis of the *Candida**glabrata* response to 5-Flucytosine: unveiling the role and regulation of the drug efflux transporters CgFlr1 and CgFlr2. Front Microbiol. 2016a;7:2045. 28066366 10.3389/fmicb.2016.02045 PMC5174090

[bib82] Paluszynski JP , Klassen R, Rohe M et al. Various cytosine/adenine permease homologues are involved in the toxicity of 5-fluorocytosine in *Saccharomyces**cerevisiae*. Yeast. 2006;23:707–15. 16845689 10.1002/yea.1387

[bib83] Pang CN , Lai YW, Campbell LT et al. Transcriptome and network analyses in *Saccharomyces**cerevisiae* reveal that amphotericin b and lactoferrin synergy disrupt metal homeostasis and stress response. Sci Rep. 2017;7:40232. 28079179 10.1038/srep40232 PMC5228129

[bib84] Pappas PG , Kauffman CA, Andes DR et al. Clinical practice guideline for the management of candidiasis: 2016 update by the Infectious Diseases Society of America. Clin Infect Dis. 2016;62:e1–50. 26679628 10.1093/cid/civ933 PMC4725385

[bib85] Pegorie M , Denning DW, Welfare W. Estimating the burden of invasive and serious fungal disease in the United Kingdom. J Infect. 2017;74:60–71. 27789254 10.1016/j.jinf.2016.10.005

[bib86] Perlin DS . Echinocandin resistance in *Candida*. Clin Infect Dis. 2015;61 Suppl 6:S612–7. 26567278 10.1093/cid/civ791 PMC4643482

[bib87] Pfaller MA , Jones RN, Castanheira M. Regional data analysis of *Candida* non-*albicans* strains collected in United States medical sites over a 6-year period, 2006-2011. Mycoses. 2014;57:602–11. 24863164 10.1111/myc.12206

[bib88] Pfaller MA , Rhomberg PR, Messer SA et al. Isavuconazole, micafungin, and 8 comparator antifungal agents' susceptibility profiles for common and uncommon opportunistic fungi collected in 2013: temporal analysis of antifungal drug resistance using CLSI species-specific clinical breakpoints and proposed epidemiological cutoff values. Diagn Microbiol Infect Dis. 2015;82:303–13. 25986029 10.1016/j.diagmicrobio.2015.04.008

[bib89] Pham CD , Iqbal N, Bolden CB et al. Role of *FKS* mutations in *Candida**glabrata*: MIC values, echinocandin resistance, and multidrug resistance. Antimicrob Agents Chemother. 2014;58:4690–6. 24890592 10.1128/AAC.03255-14 PMC4136002

[bib90] Polak A , Scholer HJ. Mode of action of 5-fluorocytosine and mechanisms of resistance. Chemotherapy. 1975;21:113–30. 1098864 10.1159/000221854

[bib91] Popp C , Ramirez-Zavala B, Schwanfelder S et al. Evolution of fluconazole-resistant *Candida**albicans* strains by drug-induced mating competence and parasexual recombination. mBio. 2019;10. DOI: 10.1128/mBio.02740-18. 10.1128/mBio.02740-18 PMC6428756 30723130

[bib92] Pujol C , Messer SA, Pfaller M et al. Drug resistance is not directly affected by mating type locus zygosity in *Candida**albicans*. Antimicrob Agents Chemother. 2003;47:1207–12. 12654648 10.1128/AAC.47.4.1207-1212.2003 PMC152535

[bib93] Ramage G , Rajendran R, Sherry L et al. Fungal biofilm resistance. Int J Microbiol. 2012;2012:528521. 22518145 10.1155/2012/528521 PMC3299327

[bib94] Reinoso-Martin C , Schuller C, Schuetzer-Muehlbauer M et al. The yeast protein kinase c cell integrity pathway mediates tolerance to the antifungal drug caspofungin through activation of slt2p mitogen-activated protein kinase signaling. Eukaryot Cell. 2003;2:1200–10. 14665455 10.1128/EC.2.6.1200-1210.2003 PMC326656

[bib95] Ricotta EE , Lai YL, Babiker A et al. Invasive candidiasis species distribution and trends, United States, 2009–2017. J Infect Dis. 2020;223:1295–302. 10.1093/infdis/jiaa502 PMC8030726 32798221

[bib96] Robbins N , Caplan T, Cowen LE. Molecular evolution of antifungal drug resistance. Annu Rev Microbiol. 2017;71:753–75. 28886681 10.1146/annurev-micro-030117-020345

[bib97] Roberts J , Bingham J, McLaren AC et al. Liposomal formulation decreases toxicity of Amphotericin b in vitro and in vivo. Clin Orthop Relat Res. 2015;473:2262–9. 25804880 10.1007/s11999-015-4232-y PMC4457755

[bib98] Rogers PD , Barker KS. Genome-wide expression profile analysis reveals coordinately regulated genes associated with stepwise acquisition of azole resistance in *Candida**albicans* clinical isolates. Antimicrob Agents Chemother. 2003;47:1220–7. 12654650 10.1128/AAC.47.4.1220-1227.2003 PMC152536

[bib99] Rosenberg A , Ene IV, Bibi M et al. Antifungal tolerance is a subpopulation effect distinct from resistance and is associated with persistent candidemia. Nat Commun. 2018;9: 2470. 29941885 10.1038/s41467-018-04926-x PMC6018213

[bib100] Rustad TR , Stevens DA, Pfaller MA et al. Homozygosity at the *Candida**albicans MTL* locus associated with azole resistance. Microbiology (Reading). 2002;148:1061–72. 11932451 10.1099/00221287-148-4-1061

[bib101] Sanglard D , Coste A, Ferrari S. Antifungal drug resistance mechanisms in fungal pathogens from the perspective of transcriptional gene regulation. FEMS Yeast Res. 2009;9:1029–50. 19799636 10.1111/j.1567-1364.2009.00578.x

[bib102] Sanglard D , Ischer F, Bille J. Role of ATP-binding-cassette transporter genes in high-frequency acquisition of resistance to azole antifungals in *Candida**glabrata*. Antimicrob Agents Chemother. 2001;45:1174–83. 11257032 10.1128/AAC.45.4.1174-1183.2001 PMC90441

[bib103] Sanglard D , Ischer F, Parkinson T et al. *Candida albicans* mutations in the ergosterol biosynthetic pathway and resistance to several antifungal agents. Antimicrob Agents Chemother. 2003;47:2404–12. 12878497 10.1128/AAC.47.8.2404-2412.2003 PMC166068

[bib104] Sanglard D , Kuchler K, Ischer F et al. Mechanisms of resistance to azole antifungal agents in *Candida**albicans* isolates from AIDS patients involve specific multidrug transporters. Antimicrob Agents Chemother. 1995;39:2378–86. 8585712 10.1128/aac.39.11.2378 PMC162951

[bib105] Satoh K , Makimura K, Hasumi Y et al. *Candida auris* sp. nov., a novel ascomycetous yeast isolated from the external ear canal of an inpatient in a Japanese hospital. Microbiol Immunol. 2009;53:41–44. 19161556 10.1111/j.1348-0421.2008.00083.x

[bib106] Selmecki A , Forche A, Berman J. Aneuploidy and isochromosome formation in drug-resistant *Candida**albicans*. Science. 2006;313:367–70. 16857942 10.1126/science.1128242 PMC1717021

[bib107] Sgherri C , Porta A, Castellano S et al. Effects of azole treatments on the physical properties of *Candida**albicans* plasma membrane: a spin probe EPR study. Biochim Biophys Acta. 2014;1838:465–73. 24184423 10.1016/j.bbamem.2013.10.015

[bib108] Shen Y , Zhang L, Jia X et al. Differentially expressed proteins in fluconazole-susceptible and fluconazole-resistant isolates of *Candida**glabrata*. Drug Discov Ther. 2015;9:191–6. 26193941 10.5582/ddt.2015.01010

[bib109] Shivarathri R , Tscherner M, Zwolanek F et al. The fungal histone acetyl transferase gcn5 controls virulence of the human pathogen *Candida albicans* through multiple pathways. Sci Rep. 2019;9:9445. 31263212 10.1038/s41598-019-45817-5 PMC6603162

[bib110] Singh SD , Robbins N, Zaas AK et al. Hsp90 governs echinocandin resistance in the pathogenic yeast *Candida**albicans* via calcineurin. PLoS Pathog. 2009;5:e1000532. 19649312 10.1371/journal.ppat.1000532 PMC2712069

[bib111] Singh-Babak SD , Babak T, Diezmann S et al. Global analysis of the evolution and mechanism of echinocandin resistance in *Candida**glabrata*. PLoS Pathog. 2012;8:e1002718. 22615574 10.1371/journal.ppat.1002718 PMC3355103

[bib112] Skrzypek MS , Binkley J, Binkley G et al. The *Candida* genome database (CGD): incorporation of assembly 22, systematic identifiers and visualization of high throughput sequencing data. Nucleic Acids Res. 2017;45:D592–6. 27738138 10.1093/nar/gkw924 PMC5210628

[bib113] Smith KJ , Warnock DW, Kennedy CT et al. Azole resistance in *Candida**albicans*. J Med Vet Mycol. 1986;24:133–44. 3014106

[bib114] Sorgo AG , Heilmann CJ, Dekker HL et al. Effects of fluconazole on the secretome, the wall proteome, and wall integrity of the clinical fungus *Candida**albicans*. Eukaryot Cell. 2011;10:1071–81. 21622905 10.1128/EC.05011-11 PMC3165447

[bib115] Steier Z , Vermitsky J, Toner G et al. Flucytosine antagonism of azole activity versus *Candida**glabrata*: role of transcription factor Pdr1 and multidrug transporter Cdr1. Antimicrob Agents Chemother. 2013;57:5543–7. 23979762 10.1128/AAC.02394-12 PMC3811237

[bib116] Suwunnakorn S , Wakabayashi H, Kordalewska M et al. *FKS2* and *FKS3* genes of opportunistic human pathogen *Candida**albicans* influence echinocandin susceptibility. Antimicrob Agents Chemother. 2018;62:e02299–17. 29358288 10.1128/AAC.02299-17 PMC5913916

[bib117] Toda M , Williams SR, Berkow EL et al. Population-based active surveillance for culture-confirmed candidemia - four sites, United States, 2012-2016. MMWR Surveill Summ. 2019;68:1–15. 10.15585/mmwr.ss6808a1 PMC6772189 31557145

[bib118] Torelli R , Posteraro B, Ferrari S et al. The ATP-binding cassette transporter–encoding gene *CgSNQ2* is contributing to the *CgPDR1*-dependent azole resistance of *Candida**glabrata*. Mol Microbiol. 2008;68:186–201. 18312269 10.1111/j.1365-2958.2008.06143.x

[bib119] Tripathi SK , Feng Q, Liu L et al. Puupehenone, a marine-sponge-derived sesquiterpene quinone, potentiates the antifungal drug caspofungin by disrupting hsp90 activity and the cell wall integrity pathway. mSphere. 2020;5. DOI: 10.1128/mSphere.00818-19. 10.1128/mSphere.00818-19 PMC6952202 31915228

[bib120] Tsai H , Krol AA, Sarti KE et al. *Candida glabrata PDR1*, a transcriptional regulator of a pleiotropic drug resistance network, mediates azole resistance in clinical isolates and petite mutants. Antimicrob Agents Chemother. 2006;50:1384–92. 16569856 10.1128/AAC.50.4.1384-1392.2006 PMC1426987

[bib121] Tsay SV , Mu Y, Williams S et al. Burden of candidemia in the United States, 2017. Clinical Infect Dis. 2020;71:E449–53. 32107534 10.1093/cid/ciaa193

[bib122] Uppuluri P , Srinivasan A, Ramasubramanian A et al. Effects of fluconazole, amphotericin b, and caspofungin on *Candida**albicans* biofilms under conditions of flow and on biofilm dispersion. Antimicrob Agents Chemother. 2011;55:3591–3. 21518839 10.1128/AAC.01701-10 PMC3122381

[bib123] Vale-Silva LA , Moeckli B, Torelli R et al. Upregulation of the adhesin gene *EPA1* mediated by *PDR1* in *Candida**glabrata* leads to enhanced host colonization. mSphere. 2016;1. DOI: 10.1128/mSphere.00065-15. 10.1128/mSphere.00065-15 PMC4863579 27303714

[bib124] vanden Bossche H , Marichal P, Odds FC et al. Characterization of an azole-resistant *Candida**glabrata* isolate. Antimicrob Agents Chemother. 1992;36:2602–10. 1482129 10.1128/aac.36.12.2602 PMC245514

[bib125] Vandeputte P , Pineau L, Larcher G et al. Molecular mechanisms of resistance to 5-Fluorocytosine in laboratory mutants of *Candida**glabrata*. Mycopathologia. 2011;171:11–21. 20617462 10.1007/s11046-010-9342-1

[bib126] Vandeputte P , Tronchin G, Larcher G et al. A nonsense mutation in the *ERG6* gene leads to reduced susceptibility to polyenes in a clinical isolate of *Candida**glabrata*. Antimicrob Agents Chemother. 2008;52:3701–9. 18694952 10.1128/AAC.00423-08 PMC2565872

[bib127] Vasicek EM , Berkow EL, Bruno VM et al. Disruption of the transcriptional regulator Cas5 results in enhanced killing of *Candida**albicans* by fluconazole. Antimicrob Agents Chemother. 2014;58:6807–18. 25182640 10.1128/AAC.00064-14 PMC4249418

[bib128] Vermitsky J , Edlind TD. Azole resistance in *Candida**glabrata*: coordinate upregulation of multidrug transporters and evidence for a pdr1-like transcription factor. Antimicrob Agents Chemother. 2004;48:3773–81. 15388433 10.1128/AAC.48.10.3773-3781.2004 PMC521908

[bib129] Vincent BM , Lancaster AK, Scherz-Shouval R et al. Fitness trade-offs restrict the evolution of resistance to amphotericin B. PLoS Biol. 2013;11:e1001692. 24204207 10.1371/journal.pbio.1001692 PMC3812114

[bib130] Walker LA , Gow NAR, Munro CA. Elevated chitin content reduces the susceptibility of *Candida* species to caspofungin. Antimicrob Agents Chemother. 2013;57:146–54. 23089748 10.1128/AAC.01486-12 PMC3535899

[bib131] Weil T , Santamaria R, Lee W et al. Adaptive mistranslation accelerates the evolution of fluconazole resistance and induces major genomic and gene expression alterations in *Candida**albicans*. mSphere. 2017;2. DOI: 10.1128/mSphere.00167-17. 10.1128/mSphere.00167-17 PMC5549176 28808688

[bib132] Whaley SG , Caudle KE, Vermitsky J et al. *UPC2A* is required for high-level azole antifungal resistance in *Candida**glabrata*. Antimicrob Agents Chemother. 2014;58:4543–54. 24867980 10.1128/AAC.02217-13 PMC4136023

[bib133] Xiang MJ , Liu JY, Ni PH et al. Erg11 mutations associated with azole resistance in clinical isolates of *Candida**albicans*. FEMS Yeast Res. 2013;13:386–93. 23480635 10.1111/1567-1364.12042

[bib134] Xu D , Jiang B, Ketela T et al. Genome-wide fitness test and mechanism-of-action studies of inhibitory compounds in *Candida**albicans*. PLoS Pathog. 2007;3:e92. 17604452 10.1371/journal.ppat.0030092 PMC1904411

[bib136] Yoo JI , Choi CW, Kim HS et al. Proteomic analysis of cellular and membrane proteins in fluconazole-resistant *Candida**glabrata*.Osong Public Health Res Perspect. 2012;3:74–78. 24159494 10.1016/j.phrp.2012.04.001 PMC3747643

[bib135] Yoo JI , Kim HS, Choi CW et al. Proteomic analysis of intracellular and membrane proteins from voriconazole-resistant *Candida**glabrata*. Osong Public Health Res Perspect. 2013;4:293–300. 24524017 10.1016/j.phrp.2013.10.001 PMC3922097

[bib137] Zhang L , Zhang Y, Zhou Y et al. Expression profiling of the response of *Saccharomyces**cerevisiae* to 5-fluorocytosine using a DNA microarray. Int J Antimicrob Agents. 2002;20:444–50. 12458139 10.1016/s0924-8579(02)00201-7

